# Brachyury expression levels predict lineage potential and axis-forming ability of *in vitro*-derived neuromesodermal progenitors

**DOI:** 10.1371/journal.pbio.3003960

**Published:** 2026-08-25

**Authors:** Anahí Binagui-Casas, Anna Granés, Alberto S. Ceccarelli, Matthew French, Filip J. Wymeersch, Rosa Portero Migueles, Jennifer Annoh, Yali Huang, Eleni P. Karagianni, Frederick C. K. Wong, Raffee Wright, Anna Sophie Brumm, Daniel Lopez Ramajo, Minoru Takasato, Sally Lowell, Osvaldo Chara, Valerie Wilson

**Affiliations:** 1 Institute for Regeneration and Repair, Centre for Regenerative Medicine, Institute for Stem Cell Research, School of Biological Sciences, University of Edinburgh, Edinburgh, United Kingdom; 2 School of Biosciences, University of Nottingham, Sutton Bonington Campus, Nottingham, United Kingdom; 3 Department of Genetics, University of Cambridge, Cambridge, United Kingdom; 4 RIKEN Center for Biosystems Dynamics Research, Kobe, Japan; 5 Department of Cell, Developmental and Integrative Biology, University of Alabama at Birmingham, Birmingham, Alabama, United States of America; 6 GSK, Stevenage, United Kingdom; 7 The Wellcome Sanger Institute, Wellcome Genome Campus, Cambridge, United Kingdom; 8 Immunology Unit, Department of Pathology and Experimental Therapy, School of Medicine, Universitat de Barcelona, Barcelona, Spain; 9 Laboratory of Molecular Cell Biology and Development, Department of Animal Development and Physiology, Graduate School of Biostudies, Kyoto University, Kyoto, Japan; 10 Instituto de Tecnología, Universidad Argentina de la Empresa, Buenos Aires, Argentina; Institute of Science and Technology Austria, AUSTRIA

## Abstract

Neuromesodermal progenitors (NMPs) produce the spinal cord and musculoskeleton in the elongating anterior-posterior axis. *In vivo*, NMPs possess dual potency, coinciding with regions co-expressing SOX2 and Brachyury (TBXT). *In vitro*, SOX2/TBXT co-expressing cells can be produced from pluripotent cells and, like their *in* *vivo* counterparts, can produce neural tube and somitic mesoderm. However, the functional characteristics of *in vitro* SOX2/TBXT co-expressing cells remain unclear, confounding comparisons with *in* *vivo* data. To address this, we developed a dual *Sox2/Tbxt* reporter mouse ESC line. SOX2/TBXT reporter-positive cells emerge *in vitro* from pluripotent populations with dynamics that mirror their appearance in the embryo. Purified SOX2/TBXT co-expressing populations can differentiate towards neurectoderm or mesoderm, including lateral mesoderm upon BMP stimulation. In gastruloids, quantitative live imaging shows that WNT or NOTCH inhibition rapidly leads to downregulation of TBXT expression and diminished axial extension. We show that clonally plated SOX2/TBXT co-expressing cells are bipotent NMPs that can also self-propagate. By combining clonal analysis with mathematical inference, we identify two thresholds of TBXT and/or SOX2 expression, switching clonal output from neural- to mesoderm-biased, and from mesoderm-biased to mesoderm-specified. Image analysis of embryonic NMPs supports a model whereby SOX2 and TBXT independently influence neuromesodermal differentiation. Thus, this *Sox2/Tbxt* double reporter cell line highlights unsuspected heterogeneity in NMPs, and together with image analysis of embryonic SOX2/TBXT levels, challenges the assumption that neuromesodermal fate choice is primarily governed by mutual antagonism between SOX2/TBXT.

## Abstract 2

Neuromesodermal progenitors (NMPs) are bipotent cells that co-express SOX2 and TBXT and generate spinal cord and musculoskeletal tissues *in vivo*. This study characterizes the functional heterogeneity of NMP-like cells, *in vitro*, and reveals how distinct SOX2 and TBXT expression thresholds govern neuromesodermal fate decisions.

## Introduction

In vertebrates, the head-to-tail (or anteroposterior, AP) body axis forms during early embryonic development. In mice, where this process is well characterised [1], a transient population of progenitors, collectively called neuromesodermal competent cells (NMCs), produces both paraxial mesoderm and neurectoderm as the AP axis elongates in post-gastrulation stages. NMC populations across vertebrates harbour cells with functional heterogeneity, including *bona fide* bipotent neuromesodermal progenitors (NMPs) [2]. Importantly, the hallmark of NMPs is their dual fate towards both neuroectodermal and mesodermal lineages, two separate germ layers, at post-gastrulation stages [3,4]. Fate mapping has shown that cells with neuromesodermal fate are found at the posterior end of various vertebrate embryo species, and this region coincides with the co-expression of the transcription factors SOX2 and Brachyury (TBXT), reviewed in Wymeersch and colleagues [1].

Our current understanding of the literature suggests that NMPs in amniotes (chick, mouse) and anamniotes (fish, frog) are distinct. In amniotes, individual bi-potent NMPs are dual neural/mesodermal fated, while in anamniotes, neuromesodermal potency is maintained in NMC populations in the tail bud, but individual cells adopt either neural or mesodermal fates [3–8]. Thus, NMCs in the neuromesodermal-fated region in different species can consist of bona fide NMPs, or of a mixture of mono-fated cells of unknown potency.

*In vitro*, FGF and WNT treatment of pluripotent stem cells generates cells that resemble *in vivo* NMC populations both phenotypically and functionally: a high percentage of cells co-express SOX2/TBXT transcription factors, and these populations can differentiate into neural and mesodermal lineages (for a list of common protocols see [1]). Indeed, at clonal level a proportion of *in vitro*-derived NMP-like cells expressing *Tbxt* (monitored with a fluorescent reporter) have dual potential for neural and mesodermal differentiation [9]. However, in these experiments, the knock-in reporter created a heterozygous null allele of TBXT [10], a condition known to affect axial development [11]. Furthermore, the expression of SOX2 in the starting population was not measured, and therefore the relationship between SOX2 and TBXT co-expression, and bipotency is unknown.

Recent single-cell RNA-seq studies have found that *in vitro* cultures harbouring NMC populations are transcriptionally heterogeneous, with varying levels of *Sox2* and *Tbxt*, as well as other NMC-specific transcripts such as *Msgn1*, *Nkx1-2,* and *Tbx6* [12–15]. One interpretation of this data is that *in vitro* NMC populations contain contaminating, non-NMP cells. Alternatively, the transcriptional heterogeneity in *Sox2* and *Tbxt*-expressing cells themselves could reflect inherent functional heterogeneity at the single-cell level given that transcription factors such as SOX2 elicit expression level-dependent function [16], and that, *in vivo*, levels of TBXT and SOX2 protein appear to correlate with variations in NMC fate [8,17,18].

Addressing whether individual *in vitro* NMP-like cells co-expressing *Sox2* and *Tbxt* are bi-potent, and how heterogeneity in SOX2 and TBXT levels dictates differentiation outcomes requires the use of cells that report on the expression of both markers. Previous reporters have employed knock-in constructs that disrupt the function of these genes [10,19] or are additive bacterial artificial chromosome transgenics where there is a risk that expression does not accurately mirror endogenous expression [20].

Here we generated a minimally disruptive mouse embryonic stem cell (mESC) line with dual *Sox2/Tbxt* gene expression reporter activity and use it to examine live cell behaviours, population heterogeneity and functional potency of *in vitro* NMP-like cells. Clonal plating demonstrates that individual *Sox2/Tbxt* dual-positive cells are *bona fide* bipotent progenitors, which can also self-propagate. Additionally, we show that rapid deceleration of axis elongation in gastruloids on WNT or NOTCH inhibition coincides with *Tbxt* downregulation, while changes in *Sox2* expression follow more slowly. We further uncover previously elusive lateral plate mesoderm potency within descendants of NMPs, an attribute shown only in bulk culture [21].

Interestingly, we find that the *Sox2/Tbxt* co-expressing population is not functionally homogeneous: the levels of *Tbxt*-GFP expression predicts clonal lineage bias towards mesoderm in individual progenitors. This intrinsic bias can be tuned by the culture conditions and cell substrates, mirroring the *in vivo* plasticity of NMC populations [8]. Investigation of this bias via mathematical inference of *in vitro* populations and image analysis of early somite stage embryos supports a model whereby TBXT and SOX2 act separately on neuromesodermal fate choice, rather than opposing one another, as suggested previously.

## Results

### 1. A novel reporter line, STR-KI, accurately reports on SOX2 and TBXT expression, and NMP differentiation trajectories *in vitro*

**Fig 1:**
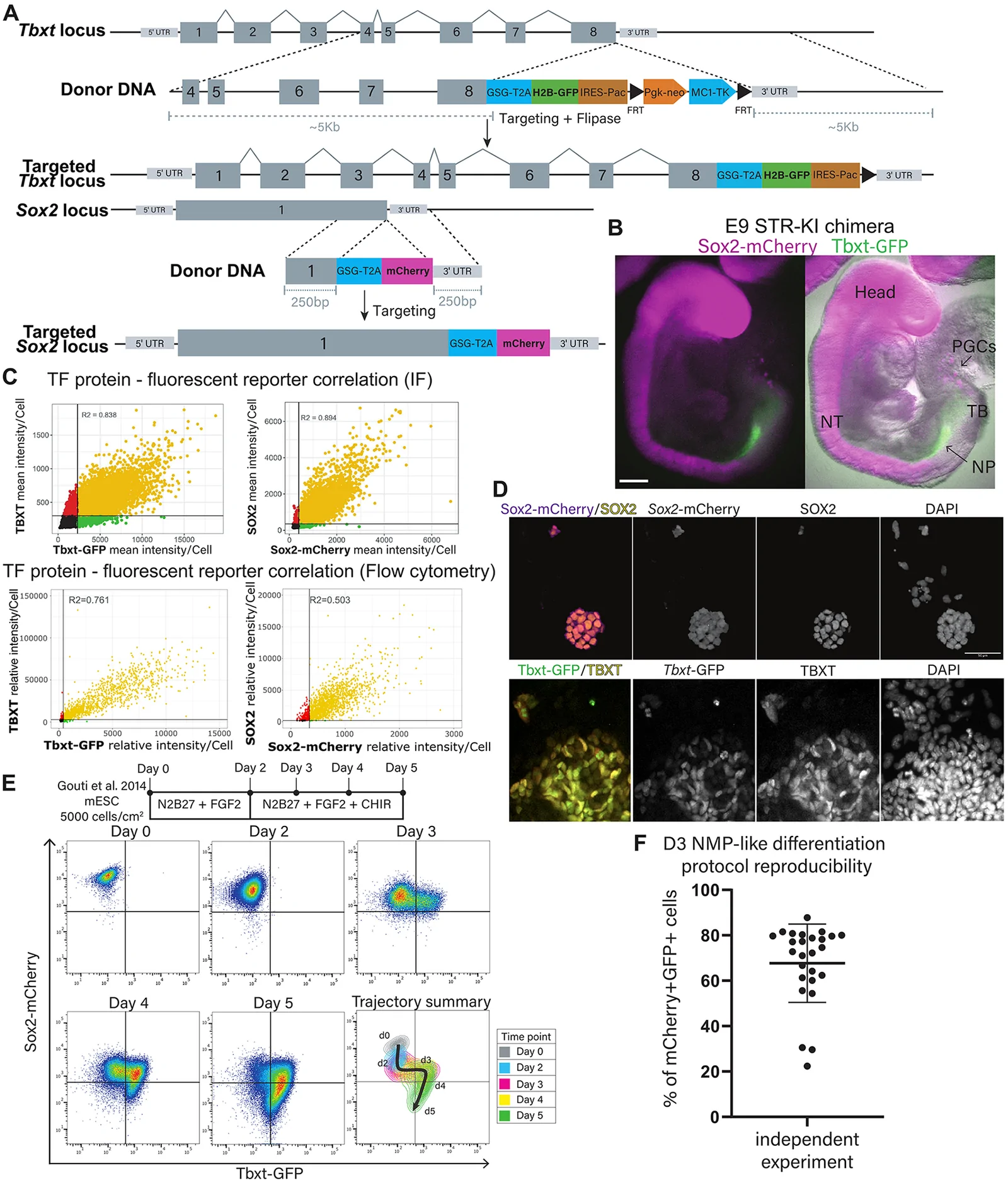
STR-KI accurately reports on SOX2 and TBXT expression, and NMP differentiation trajectories *in vitro.* **(A)** Schematic illustration of the targeting strategy to knock-in GFP and mCherry in the *Tbxt* and *Sox2* loci, respectively. **(B)** Embryonic day 9 (E9) STR-KI chimaera with high STR-KI contribution shows appropriate localisation of fluorescent reporter expression: high mCherry (magenta) in the prospective brain, neural tube (NT) and primordial germ cells (PGCs). GFP (green) expression marks the notochordal plate (NP) and tail bud (TB). Scale bar = 500 μm. **(C)** Correlation between immunofluorescent staining for SOX2 or TBXT, and their respective reporter protein in individual cells derived from *in vitro* NMP differentiation assays. Top panel shows correlations assayed by immunofluorescent confocal imaging and bottom panel by flow cytometry. **(D)** Immunofluorescence images of differentiated STR-KI colonies following NMP-like differentiation [25]. Scale bar = 50 μm. **(E)** Representative example from a flow cytometry time-course analysis of STR-KI cells along the differentiation protocol (above), from mESCs (day 0) via NMP-like intermediate (at day 3) to day 5 (*n* = 4). A trajectory summary highlights population dynamics. **(F)** Quantification of the reproducibility of STR-KI mESC differentiation to day 3 NMP-like cells shows the percentages of Sox2^mCh^/Tbxt^GFP^ double-positive cells for a cohort of independent experiments (*n* = 23; Mean ± SD: 67.42 ± 18.28%). Data for Fig 1F: https://doi.org/10.5281/zenodo.21708690.

To characterise the dynamics of NMC emergence from mESCs, we constructed a 2A-peptide-linked dual reporter: SOX2::mCherry; TBXT::GFP, hereafter named SOX2-TBXT Reporter Knock-In (STR-KI). CRISPR knock-in transgenesis was used to create heterozygous knock-in alleles that replace the stop codon of the endogenous gene loci with T2A/P2A ‘self-cleaving’ (ribosome-skipping [22]) peptide, retaining the *Tbxt* and *Sox2* open reading frames in their entirety [23]. Since even partial loss of TBXT or SOX2 function affects differentiation [11,16,19,24], we employed this strategy to minimise the chance of adverse phenotypic effects and maximise the faithfulness of the reporters (Fig 1A). Chimeric embryos containing high contribution from STR-KI mESCs showed a normal phenotype, demonstrating that the gene targeting was indeed minimally disruptive to SOX2 and TBXT protein function (Figs 1B and S1C). Furthermore, the fluorescent reporters in pre-streak and somite stage embryos recapitulated the *in vivo* expression pattern of TBXT and SOX2 in mesodermal and neural lineages, respectively (Figs 1B and S1C) [8]. In pre-streak chimaeras, SOX2::mCherry (Sox2^mCh^ hereafter) was expressed throughout the epiblast, whereas TBXT::GFP (Tbxt^GFP^ hereafter) expression was undetectable (S1C Fig). By early somite stages onwards, Sox2^mCh^ was predominantly restricted to neural tissues, and to presumptive primordial germ cells at the base of the allantois, whereas Tbxt^GFP^-expressing cells were observed in the node, notochord and nascent mesoderm at the posterior end (Figs 1B and S1C). Differentiation of STR-KI ESCs into NMP-like cells, following an established protocol [25] produced mCherry and GFP double-positive cells (Sox2^mCh^pos/Tbxt^GFP^pos) (Fig 1C–1F). Immunostaining and quantification of transcription factor (TF) intensity in these *in vitro* differentiated cells showed that the reporters faithfully co-localised with endogenous SOX2 and TBXT protein (Figs 1C, 1D, and S1B). For both reporters, we also observed a positive correlation between the levels of reporter and endogenous protein in immunofluorescence followed by confocal imaging (SOX2-mCherry *R*^2^ = 0.894, TBXT-GFP *R*^2^ = 0.838), and intracellular staining followed by flow cytometry (SOX2-mCherry *R*^2^ = 0.503, TBXT-GFP *R*^2^ = 0.761) (Fig 1C). We therefore adopt the working hypothesis that GFP and mCherry levels reflect TBXT and SOX2 protein production, respectively, although differential posttranscriptional modifications affecting protein levels cannot be excluded.

We next tracked the reporters’ dynamics over a time course of NMP differentiation (Fig 1E). Briefly, this entails differentiation of ESC to an epiblast-like state, followed by treatment with the WNT agonist CHIR99021 (CHIR) and FGF2 from day (d) 2 onwards [25]. Cells appeared to move along a differentiation trajectory from Sox2^mCh^high pluripotent ESC to a Sox2^mCh^low, putative epiblast state, followed by appearance of Sox2^mCh^pos/Tbxt^GFP^pos (double positive cells) within 3 days. D3 differentiated cells exhibited a spectrum of Tbxt^GFP^ levels before exiting the double-positive state towards Sox2^mCh^-negative (neg)/Tbxt^GFP^high mesoderm from d4. Consistent with previous reports, STR-KI ESC differentiation reproducibly resulted in the production of putative NMCs co-expressing mCherry and GFP at a rate of ~67% of the population by d3 (Fig 1F).

### 2. Expression dynamics of *Sox2* and *Tbxt* *in vitro* matches NMP emergence *in vivo*

A previous report demonstrated that *Sox2* expression in the caudal lateral epiblast is activated de novo in mouse via its N1 enhancer activity [26], corresponding approximately to the time at which NMCs are a detectable cell population [8]. Superficially, this would suggest that *Sox2* is secondarily activated in the *Tbxt*-expressing caudal lateral epiblast, contrasting with our observation of the acquisition of *Tbxt* in *Sox2*-expressing cells *in vitro* (Fig 1E). We therefore examined the origin of NMPs in the epiblast.

**Fig 2:**
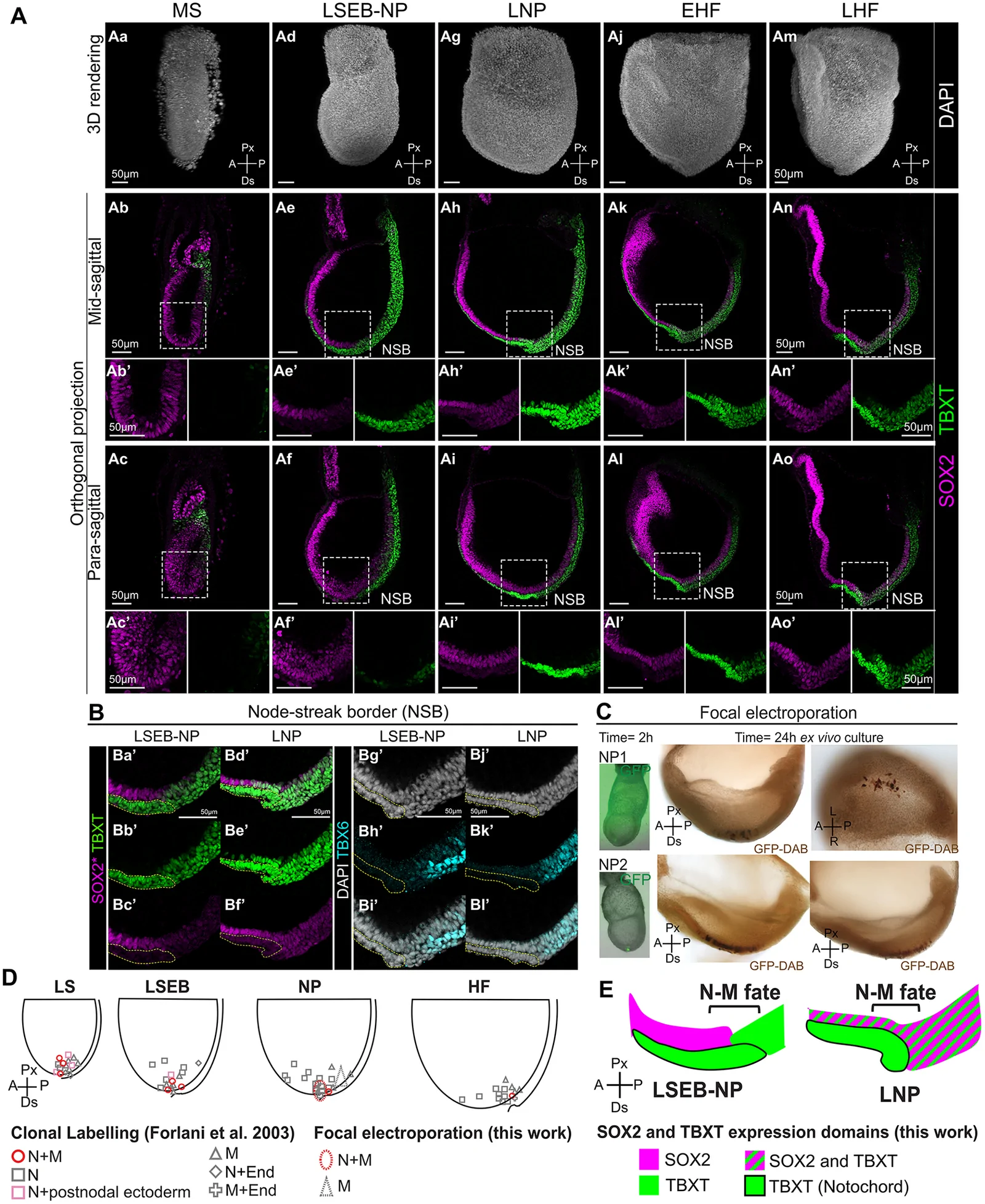
NMPs emerge *in vivo* from a pre-existing SOX2+TBXT- population. **(A)** Immunofluorescent detection of SOX2 and TBXT in the fate-mapped region (Node-streak border, NSB – dashed line boxed) over developmental time. Upper row: DAPI-stained 3D renderings of mouse embryos (dissected on embryonic days E6.5 (midstreak, MS), E7.0 (late streak early bud, LSEB), E7.5 (late neural plate, LNP) and E8.0 (early and late head fold, EHF, LHF); as staged in [27]). Lower rows: Orthogonal projections of wholemount immunostained mouse embryos for SOX2 (magenta) and TBXT (green), showing mid- and para-sagittal optical sections of the node area (projections represent 8 µm across the Z axis). All scale bars are 50 µm. **(B)** Left: Immunofluorescent detection of SOX2 (magenta) and TBXT (green) in LSEB-NP and LNP embryos as depicted in A (dashed line boxes), but with the SOX2 field under enhanced exposure (SOX2*). Right: Immunofluorescent detection of TBX6 (cyan) in LSEB-NP and LNP embryos, and counterstained with DAPI (grey). As a reference, the node/notochord is circled by dashed lines. **(C)** Fate mapping at neural plate (NP) stage via focal electroporation of a ubiquitously expressed CAG-GFP transgene into cells anterior to the node tracks descendants into the neural plate and anterior caudal lateral epiblast (*n* = 2). Equivalent electroporation in the primitive streak (*n* = 3) produces descendants in the mesoderm (data summarised in D). **(D)** Focal electroporations superimposed on data from [28], where cells in or near the node at late streak (LS) to head fold (HF) stages were prospectively mapped to segments of the axis. Data has been re-displayed to indicate clones producing descendants in neurectoderm and/or mesoderm. **(E)** Summary scheme of data in A–D. Tracing of LSEB-NP and NP stage node region showing expression of SOX2 and TBXT in epiblast and nascent notochord. Expression is non-overlapping at the midline in LSEB-NP transition-stage embryo, while in LNP-stage embryo onwards, SOX2+ TBXT+ cells are evident. The anteroposterior extent of NM fate in the epiblast, extracted from diagrammatic representations in Forlani and colleagues at each stage is indicated, showing N-M fate is initially localised in the SOX2+ dorsal node region, which later becomes SOX2+TBXT+. Px, proximal; Ds, distal; A, anterior; P, posterior; L, left; R, right. All scale bars are 50 µm.

For this, we first analysed SOX2 and TBXT immunofluorescence in embryos staged between mid-streak and late headfold [27] (Fig 2A), including the neural plate (also known as pre-headfold) stage at which TBXT+SOX2+ putative NMPs emerge [8]. Up to early neural plate stage, SOX2 single-positive cells were located anterior to the node, while cells posterior to the node expressed only TBXT. Double-positive cells emerged around the node and just posteriorly to it during neural plate stage. These constituted most of the cells at the posterior node from late neural plate (LNP) stage onwards, as seen by staining for post-nodal TBX6 in this same regions, which delineates mesoderm and the primitive streak (Fig 2B and S1 Movie). Comparison of this staining pattern with focal electroporations at neural plate stage (*n* = 2 anterior to node, *n* = 3 posterior to node, Fig 2C and 2D) and the integration of our data with previously published *in vivo* single cell clonal lineage tracing around the node [28] (Fig 2D) shows that before neural plate stages, cells with dual neuromesodermal fate are located anterior to the node, while those posterior to it are exclusively mesoderm-fated. Thus, NMPs in the embryo emerge from the SOX2+ cell population anteriorly to the node and subsequently acquire TBXT expression (Fig 2E). This indicates that the acquisition of TBXT expression by SOX2+ cells in NMP differentiation conditions *in vitro* mimics the *in vivo* ontogeny of NMPs.

### 3. *In vitro* derived Sox2^mCh^/Tbxt^GFP^ co-expressing cells are NMCs

**Fig 3:**
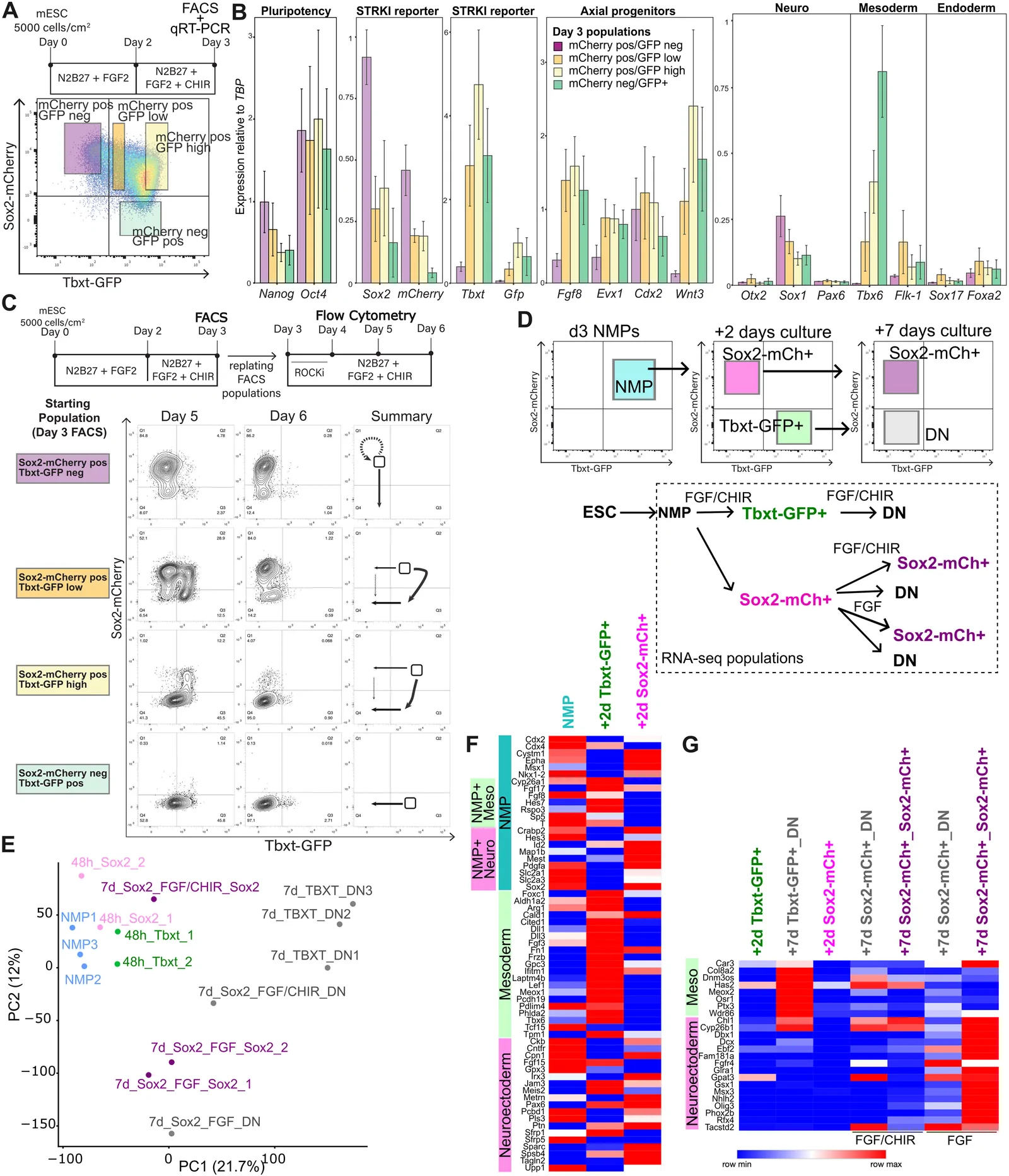
Gene expression and lineage potential analysis of Sox2^mCh^/Tbxt^GFP^ fluorescent subpopulations during differentiation. **(A)** Differentiation scheme and representative gating strategy for FACS and further qRT-PCR analyses performed at day 3 of the differentiation protocol. **(B)** qRT-PCR gene expression analysis of the populations in A. *n* = 5 independent differentiation experiments. **(C)** Top: Post-d3 differentiation scheme in which populations in A were isolated by FACS, replated and further differentiated for an additional 3 days. Bottom: Representative flow cytometry graphs of one time-course experiment (*n* = 3, see S2C Fig), following the Sox2^mCh^ and Tbxt^GFP^ expression dynamics over three more days. A summary of the lineage outcomes in three experiments over time is provided. The thickness of arrows represents the relative contribution to the indicated trajectory, with dashed arrows indicating a presumed trajectory based on the proportions of cells at the end of the experiment. **(D)** Experimental schematic for RNA-seq analysis of NMP-derived populations. Top: schematics of the sorting strategy: day 3 NMPs are sorted and cultured for two days and resorted and cultured for seven additional days under FGF/CHIR conditions, generating Tbxt^GFP^pos and Sox2^mCh^pos subpopulations at day +2, and Sox2^mCh^pos and double-negative (DN) subpopulations at day +7. Bottom: summary of the differentiation routes, culture conditions and populations collected for RNA-seq. For NMP *n* = 3, for +2d Sox2+ and Tbxt+ *n* = 2, for Tbxt+ derived DN *n* = 3, for Sox2+ derived DN *n* = 1 per culture condition (FGF or FGF/CHIR), for Sox2+ derived Sox2+ *n* = 2 in FGF and 1 in FGF/CHIR. **(E)** PCA of RNA-seq transcriptomes from all sorted populations. Each dot represents an independent biological sample, coloured by population identity. PC1 (21.7%) and PC2 (12%) separate samples by differentiation state. **(F)** Heatmap of lineage-associated gene expression across NMPs and 48h sorted populations (Tbxt^GFP^pos and Sox2^mCh^pos). Genes are grouped by lineage identity: NMP, NMP+Mesoderm, NMP+Neuro, Mesoderm, and Neuroectoderm. **(G)** Heatmap of mesodermal and neuroectodermal marker gene expression across 48 h and day 7 sorted populations, showing transcriptional divergence between Tbxt^GFP^pos -and Sox2^mCh^pos-derived cells and the effect of FGF/CHIR vs. FGF treatment on day 7 Sox2^mCh^pos and DN populations. Expression values are row-normalised (row min to max). Data for Fig 3B–3G: https://doi.org/10.5281/zenodo.21708690.

To characterise the cell populations emerging along the ESC-to-NMP-like cell trajectory, we probed the gene expression profiles of various subpopulations for diagnostic marker genes from d2 to d4 via qRT-PCR (Figs 3A, 3B, S2A, and S2B). Subpopulations were delineated based on the intensities of mCherry and GFP reporter detection in FACS. Comparison of mRNA expression levels of endogenous *Sox2* and *Tbxt* with their respective reporters *mCherry* and *GFP* showed that in all sorted populations, mRNA expression reflected endogenous gene expression levels. The levels of *Sox2* and *Tbxt* mRNAs exceeded those of the corresponding reporters, as expected, since the reporter allele, which transcribes both endogenous mRNA and reporter, is heterozygous (Fig 3B). Interestingly, the putative NMC population (Sox2^mCh^pos/Tbxt^GFP^pos) displayed relatively constant Sox2^mCh^ fluorescence but a spectrum of Tbxt^GFP^ expression. Since the levels of Tbxt^GFP^ in the double-positive population increased before the appearance of Tbxt^GFP^ single-positive mesoderm cells, we hypothesised that low and high Tbxt^GFP^ populations might represent different states in the wider NMC population. Specifically, high Tbxt^GFP^-positive cells might be further along a mesoderm differentiation trajectory compared to Tbxt^GFP^-low cells [8,17,18].

To test this, we divided the NMC-like population into low-TBXT (Sox2^mCh^pos/Tbxt^GFP^low) and high-TBXT (Sox2^mCh^pos/Tbxt^GFP^high) expressing subpopulations, where SOX2 levels were relatively constant. We analysed subpopulations based on marker gene expression: pluripotency-associated *Nanog* and *Oct4*; axial progenitor *Fgf8*, *Evx1*, *Cdx2* and *Wnt3a*; paraxial mesoderm *Tbx6;* lateral plate mesoderm (LPM) *Flk-1*; and neural-associated *Otx2, Sox1* and *Pax6*. Although both low-TBXT and high-TBXT populations expressed similar levels of NMC markers, high-TBXT cells were enriched for mesodermal markers such as *Wnt3a* and *Tbx6*, while expression of neural genes like *Sox1* were low. The absence of *Sox2*, coupled with high expression of *Tbx6* and *Flk-1* in both Sox2^mCh^neg/Tbxt^GFP^pos and Sox2^mCh^neg/Tbxt^GFP^neg (double negative) populations, together with the downregulation of NMC/caudal markers like *Fgf8* and *Wnt3a* from d3 to d4, suggests that these two populations represent nascent and differentiated mesoderm, respectively (S2A and S2B Fig). Finally, the expression of endoderm markers *Sox17* and *Foxa2* was universally low, suggesting negligible fractions of contaminant non-NMC cells. Thus, gene expression analysis in populations undergoing differentiation confirms that our double reporter cell line faithfully recapitulates endogenous gene expression and can be used to purify neural, mesodermal and NMC-like cell populations based on Sox2^mCh^ and Tbxt^GFP^ expression levels (Figs 3B, S2A, and S2B). Furthermore, this analysis supports the idea that high levels of Tbxt^GFP^ mark a more mature mesodermal state than low Tbxt^GFP^ levels.

To test whether the apparently dynamic changes in Sox2^mCh^ and Tbxt^GFP^ cell populations during NMP differentiation (Fig 1E) represent one or more lineage trajectories, the lineage potentials of sorted subpopulations at d3 of NMP differentiation were assayed by FACS over 3 further days in continuous FGF and CHIR, which are permissive for both neural and mesodermal potential [9,25] (Figs 3C and S2C). Most (>80%) Sox2^mCh^ single-positive cells remained single-positive. Together with the enrichment of *Sox1* and *Nanog* in the starting population (Fig 3B), this suggests a neural and/or epiblast identity of Sox2^mCh^ single-positive cells [29]. A small proportion of the progeny lost expression of both reporters, likely representing further maturation, since SOX2 is downregulated during neural differentiation. Sox2^mCh^pos/Tbxt^GFP^low cells produced Sox2^mCh^ single-positive cells by d6, but also a recapitulation of the pattern shown in Fig 1E: emergence of Sox2^mCh^pos/Tbxt^GFP^high cells, followed by a high proportion of Sox2^mCh^neg/Tbxt^GFP^pos and later double-negative cells. This suggests that Sox2^mCh^pos/Tbxt^GFP^low NMCs produce both neural and mesodermal cells. Interestingly, Sox2^mCh^pos/Tbxt^GFP^high cells produced few Sox2^mCh^ single-positive cells and followed a predominantly mesoderm trajectory, producing Sox2^mCh^neg/Tbxt^GFP^pos and then double-negative cells. Finally, Sox2^mCh^neg/Tbxt^GFP^pos populations only produced double-negative cells. Together with its enrichment in expression of mesoderm markers, it suggests this is a mesoderm-committed population. Thus, using our dual reporter, we describe a lineage trajectory in which a largely self-sustaining SOX2-positive population generates a SOX2-positive;TBXT-low dual-potency population, which progresses through a mesoderm-biased SOX2-positive;TBXT-high state towards a nascent mesodermal TBXT-high and, finally, a double-negative state (Fig 3D, see summary). This indicates that the TBXT-high NMC population is predisposed towards mesoderm differentiation. Therefore, within the Sox2^mCh^pos/Tbxt^GFP^pos double-positive NMC population, there are cells with apparent differing competence to differentiate to neural or mesodermal outcomes.

To investigate the identity of the later populations, we next characterised intermediate progenitors and their main derivative populations by bulk RNA sequencing (Fig 3D and 3E). We sorted d3 NMC-like Sox2^mCh^pos/Tbxt^GFP^pos cells and cultured for two further days to capture their descendant Sox2^mCh^pos/Tbxt^GFP^neg and Sox2^mCh^neg/ Tbxt^GFP^pos populations (Fig 3F). We then cultured these cells for a further 7 days, sorting these final populations to their constituent Sox2^mCh^/Tbxt^GFP^ phenotypes. We found that culturing Sox2^mCh^pos cells over seven days in FGF/CHIR did not enrich for a neural phenotype and therefore included an FGF-only condition for this population (Fig 3G). A principal component analysis (PCA) plot showed that replicate samples clustered together, and that Sox2^mCh^pos and Tbxt^GFP^pos cells cluster apart from each other and from the starting NMC-like population. The Tbxt^GFP^pos-derived cells plus 7 days clustered far from the undifferentiated samples, as did the FGF-treated Sox2^mCh^pos-derived samples. In contrast, FGF/CHIR-treated Sox2^mCh^pos-derived samples did not diverge far along either PC axis, suggesting limited differentiation (Fig 3E).

We next examined a set of mesoderm- and neural-specific markers for E8–8.5 recently differentiated mesoderm and neurectoderm. These were drawn from the top 50 differentially expressed genes (DEGs) between a derivative cell and its progenitor. We validated the mesoderm or neural character of DEGs between 2d samples and NMPs via comparison with a mouse single-cell RNA-seq atlas [30]. To assess the neural or mesodermal character of 7d samples, we manually curated the top 50 DEGs between 7d populations and their progenitors through searching E9.5–10.75 images and their annotations in the Mouse Genome Informatics database [31]. During the 2 days after NMP cell plating, cells in the Tbxt^GFP^pos compartment had lost NMP-specific expression at the expense of early mesoderm differentiation (Fig 3F). A further 7 days of culture led to most of the cells becoming double negative (DN) and expressing relatively mature mesoderm markers (Fig 3G). In contrast, day 2 Sox2^mCh^pos cells retained some NMP markers while enriching some neural markers (Fig 3F). Further culture in FGF enriched for neural differentiation markers, whereas FGF/CHIR did not result in neural marker upregulation (Fig 3G). These results support our starting hypothesis that TBXT^GFP^pos cells are mesoderm-fated, eventually downregulating TBXT as they pass from nascent to mature mesoderm, confirming that the predominant DN population in TBXT^GFP^pos-derivatives is mesoderm. We additionally suggest that WNT pathway activation in Sox2^mCh^pos cells is incompatible with progression to a more differentiated neural phenotype, while FGF treatment alone permits neural differentiation.

Previous reports have shown that treatment of pluripotent cells with FGF2 and CHIR leads to the expression of both paraxial and lateral plate mesoderm (LPM) markers [14,15]. *In vivo*, while homotopic grafts of NMCs overwhelmingly give rise to paraxial mesoderm [8,32], heterotopic grafts to the posterior primitive streak lead to LPM differentiation [21]. This suggests that NMPs may have LPM potency, although it cannot be excluded that this LPM arose from non-NMCs within the graft. To directly test the LPM-potency of NMCs, we examined the potential of different FACS-enriched populations at d3 of the NMP protocol upon BMP treatment, which induces LPM differentiation in bulk NMP cultures (S2D Fig) [21]. In Sox2^mCh^pos/Tbxt^GFP^neg and Sox2^mCh^pos/Tbxt^GFP^low populations, we observed not only FLK-1 protein (a lateral plate/endothelial cell marker) labelling in colonies but also the formation of endothelial sheets and primitive tubes similar to those seen in classical endothelial assays [33] (S2E Fig). These populations were markedly different in their potential to generate and maintain SOX2+ and TBXT+ populations, since Sox2^mCh^pos/Tbxt^GFP^low cells are able to generate and maintain both while only SOX2+  cells seem to remain in culture from the Sox2^mCh^pos/Tbxt^GFP^neg population. Surprisingly, NMP populations characterised by high Tbxt^GFP^ expression produced virtually no FLK-1 positive cells by the end of the culture and showed no TBXT and SOX2 staining, suggesting that high Tbxt expression at the NMP stage is incompatible with subsequent endothelial fate acquisition under these conditions. These findings suggest that the most immature epiblast-like or NMC populations (marked by no or low-Tbxt expression, respectively) can respond to BMP4 to produce LPM whereas Tbxt-high NMC populations might be already committed to a paraxial fate, a finding supported by the enrichment of paraxial mesoderm markers (e.g., *Dll1, Dll3, Tbx6, Meox1, Tcf15, Lef1, Cited1, Ald1a2*), in Tbxt^GFP^pos populations. Nevertheless, our *in vitro* results support the idea that *in vivo* Tbxtlow NMCs themselves, and not contaminating populations, can contribute to both paraxial and lateral mesoderm if provided with permissive environmental cues.

### 4. Live imaging of STR-KI cells uncovers a role for TBXT in WNT and NOTCH-driven axis elongation

*In vivo*, wildtype TBXT levels are not only critical for NMP differentiation, but also to maintain axial elongation [1]. However, the dynamic relationship between TBXT, SOX2 and axial elongation remains untested. To determine in real time the relationship between reporter expression, axial elongation and factors known to affect both aspects, we next used live-tracking of STR-KI reporter expression. For this, we tracked Sox2^mCh^ and Tbxt^GFP^ expression *in vitro* in a standard gastruloid model of axis elongation, in which aggregates of mESCs grown in N2B27 remain SOX2 positive/TBXT negative until 48 hours post-aggregation (hpa), then exit pluripotency [34,35]. At 48 hours, a 24-hour pulse of CHIR is used to activate WNT globally and ensure robust symmetry breaking. WNT activation transiently induces TBXT expression in all cells [35], before it resolves into polarised expression at the posterior pole by 72 hpa and is then followed by elongation. We first compared the dynamics of mesoderm differentiation in STR-KI-gastruloids with previous reports (S2F Fig). After treatment with CHIR, most cells became Tbxt^GFP^pos by 72 hpa, and down-regulated Sox2^mCh^. In the absence of CHIR, STR-KI cells remained Sox2^mCh^high, but a small proportion of cells began to express Tbxt^GFP^. These trends were maintained at the population level by 96hpa (S2F Fig). We also observed downregulation of Sox2^mCh^, symmetry breaking and polarisation of Tbxt^GFP^ to the posterior growing axis only upon the CHIR pulse (S2G Fig). We conclude that our STR-KI reporter can generate gastruloids with similar timings to cell lines in other studies (including a TBXT-GFP reporter line, [35]) and thus it is ideally suited for studying TF dynamics within them.

**Fig 4:**
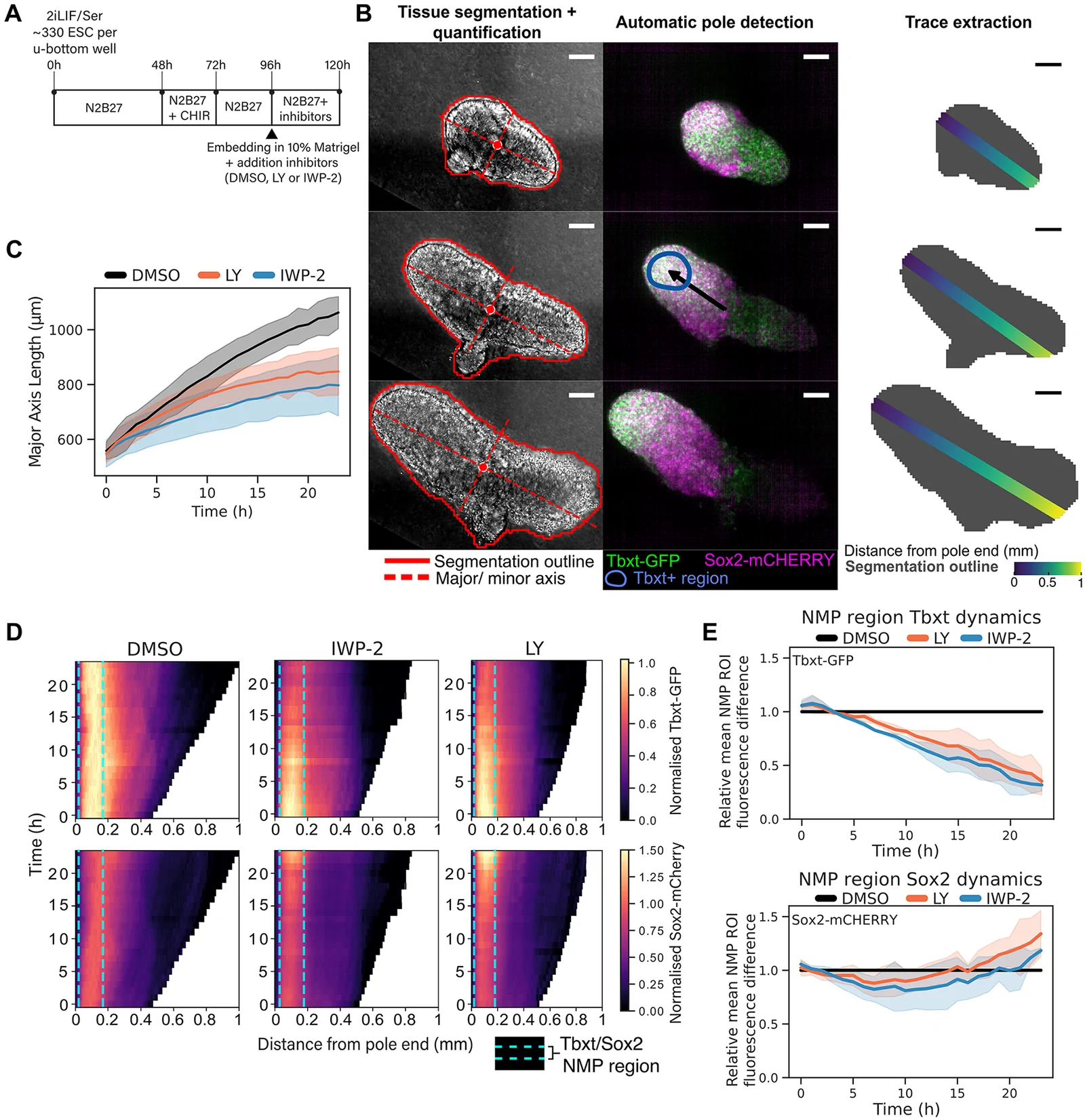
STR-KI cell analysis indicates that Tbxt-expressing cells are responsible for WNT and NOTCH-driven gastruloid elongation. **(A)** Experimental setup for the generation of STR-KI gastruloids in 10% matrigel at 96 hours with DMSO, WNT signalling inhibitor IWP2 (2.5 µM) or Notch signalling inhibitor LY411575 (150 nM). Gastruloids were imaged on a confocal microscope every hour for 24 h after embedding. Examples of timelapses can be seen in S2–S6 Movies. **(B)** Quantification pipeline of 3D gastruloid confocal image stacks. Blue circle = high Tbxt^GFP^ region (top 90% in the gastruloid, see methods). All scale bars indicate 100 µm. **(C)** Axial length after treatment of gastruloids with IWP-2 or LY411575. **(D)** Heatmaps showing the average Tbxt-GFP or Sox2-mCherry signal per binned distance from the pole along time post Matrigel embedding. **(E)** Average difference in fluorescence signal of the Sox2/Tbxt region between control and treated conditions. *N* = 3 independent experiments. Total gastruloids analysed: DMSO = 35, LY = 38, IWP-2 = 41. Shaded areas indicate the mean ±0.95 confidence intervals, calculated by *x̄* ± z*(*σ*/sqrt(*n*)). Data for Fig 4B–4E: Data file 1–3, https://doi.org/10.5281/zenodo.21676159.

We next assessed how the manipulation of WNT and NOTCH signalling pathways, which regulate mesoderm differentiation from NMPs [8,36], impact gastruloid axial elongation. We treated elongating gastruloids (96 hpa) with either WNT or NOTCH inhibitors (IWP-2 [37] and LY411575 (LY) [38], respectively) and live imaged them every hour for 24 hours to monitor major axis length and reporter expression (Fig 4A and 4B). Both WNT and NOTCH inhibition decelerated major axis growth (elongation) starting from as early as 2–3 h post-treatment (Fig 4C).

To determine how reporter expression responded to WNT or NOTCH inhibition during this period, whole gastruloid tissues were segmented and traces extracted along the extending long axis marked at the presumptive posterior end by Tbxt^GFP^ expression. Treatment with IWP2 or LY rapidly decreased Tbxt^GFP^ signal in the NMP region (within ~2–3 h). In contrast, Sox2^mCh^ signal in this region increased after ~10–15 hours following an initial minor decrease compared to the DMSO control (Fig 4D), which could be the result of a delayed overproduction of neural cells or an overall Sox2 level increase on specification to neural identity. These changes in gene expression coincide in time with growth deceleration (Fig 4D and 4E). Thus, the rapid Tbxt^GFP^ signal downregulation coupled with slowdown of axis elongation in response to WNT or NOTCH inhibition suggests AP axis elongation is driven predominantly by *Tbxt-*expressing cells.–.

### 5. Clonal tracing of *in vitro* derived NMCs reveals lineage bias of NMPs dependent on matrix and TBXT levels

**Fig 5:**
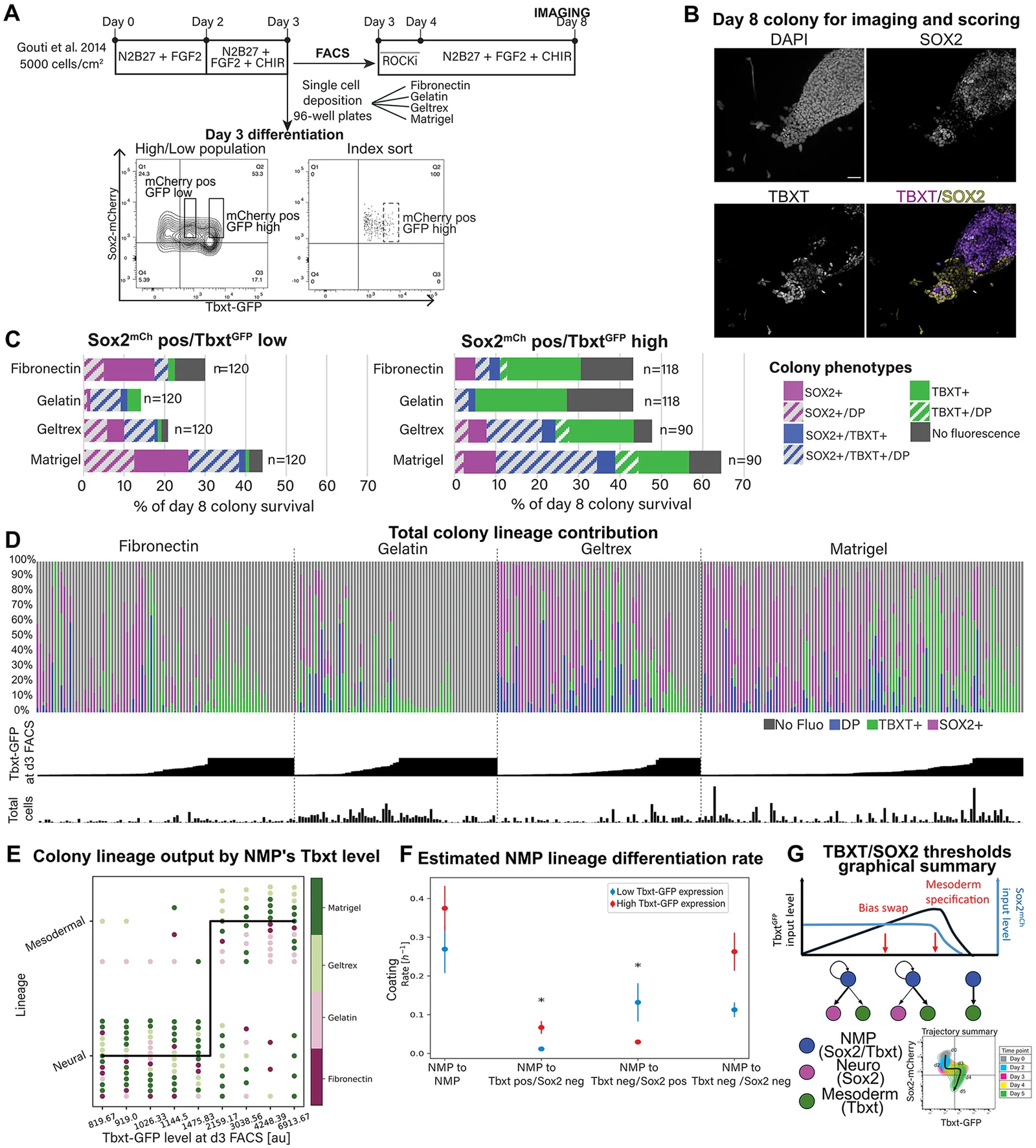
*In vitro*-derived *Sox2*/*Tbxt* co-expressing cells are bipotent at clonal level and their lineage output is determined by *Tbxt* expression. **(A)** Differentiation protocol scheme and representative gating strategy for FACS and index sorting according to Sox2^mCh^ and Tbxt^GFP^ expression. Cells were individually seeded into different substrates and cultured a further five days. Colony lineage output was assessed by immunofluorescence of endogenous SOX2 and TBXT proteins, after which the imaging data was segmented for individual cells in colonies and scored for their lineage output. **(B)** Example of a colony on gelatine at the end of the experiment immunostained for SOX2 and TBXT. **(C)** Summary of the total lineage output of colonies derived from single-cell deposition of sorted Sox2^mCh^pos;Tbxt^GFP^high or Sox2^mCh^pos;Tbxt^GFP^low NMP cells. Colonies were immunostained for endogenous SOX2 and TBXT proteins, and analysed at 8 days post-sorting. Stripes indicate colonies that include double-positive putative self-renewing NMPs alongside single-positive cells, and colours denote their derivatives: SOX2pos (magenta), TBXTpos (green), a mixture of the two single-positive cell types (blue), or only double-negative cells (grey) were detected, with n being the number of colonies scored for each substrate. **(D)** Overview of all individual colonies scored on their percent lineage output, classified by substrate and ordered by Tbxt^GFP^ level (trace below clonal composition chart) at the time of FACS (day 3). The total number of cells (lower trace) per colony ranged from 2 to 5,335 cells. Note that the plateau in GFP level is arbitrary and corresponds to progenitors that were gated for Sox2^mCh^pos;Tbxt^GFP^high but not index sorted, and demarcates a mesoderm commitment threshold. Colours indicate percentage of colony with indicated phenotype. **(E)** Individual colony lineage output as a function of the initial Tbxt^GFP^ levels in the founder NMP. Each dot represents one colony, coloured by substrate used: Matrigel (dark green), Geltrex (light green), gelatine (pink) and fibronectin (fuchsia). The data was segregated in bins of equal number of colonies and the Tbxt-GFP level on the horizontal axis is the average of input levels per bin. The black line shows a step-function fit to the data, indicating a neural-to-mesodermal lineage transition at ~1,745 Tbxt-GFP [AU] (bias switch at the vertical). **(F)** Mathematical estimation of fate in index-sorted NMPs. The rate indicates whether single founder NMPs were above (red) or below (blue) the initial Tbxt level threshold of 1,745 arbitrary units (au) as function of their colony output at the end of the experiment. The dots and the error bars correspond to the mean and the standard error of the mean, respectively. Asterisks indicate significant differences obtained after applying a Mann–Whitney statistical test (*p* < 0.05). **(G)** ‘Two-threshold model’ summarising our clonal data. Data for Fig 5C and 5D: https://doi.org/10.5281/zenodo.21708690; for 5E, 5F: https://doi.org/10.5281/zenodo.21650680.

Our findings at the bulk level suggest that differences in *Tbxt* levels influence lineage outcomes in Sox2/Tbxt co-expressing (NMC) subpopulations (Fig 3D). However, these experiments did not distinguish between neural or mesoderm lineage biases across the whole *in vitro* NMC-like population or a subset of already lineage-specified cells expressing the very highest or lowest reporter levels. To distinguish these possibilities, we sorted Sox2^mCh^pos;Tbxt^GFP^pos cells by FACS into Tbxt low and high gates and deposited single cells into 96-well plates (Fig 5A). We measured their differentiation outcome in FGF/CHIR, which supports both neural and mesodermal differentiation (Fig 3D). A variety of substrates has been used in different NMC-generating protocols, and the influence of these is currently unclear. We hypothesised that in addition to *Tbxt* expression levels, extracellular matrices might also impact cell fate decisions. We therefore compared clonal outcomes on four commonly used matrices: fibronectin, gelatine, Geltrex and Matrigel. We then correlated this information with lineage output using immunofluorescence and high-throughput microscopy to quantify the SOX2/TBXT cellular phenotype in descendant cells in each well after five days (Fig 5A and 5B).

We first considered the characteristics of all clones, sorted into ‘low’ and ‘high’ GFP gates as in Fig 3A. In all clonal conditions tested, Sox2^mCh^pos;Tbxt^GFP^pos cells produced single-positive neural or mesodermal descendants, as well as double-positive cells, confirming that these cells are NMPs. Sox2^mCh^pos;Tbxt^GFP^low and Sox2^mCh^pos;Tbxt^GFP^high input cells showed distinct lineage output profiles. Sox2^mCh^pos;Tbxt^GFP^high cells gave rise to a greater proportion of Tbxt^GFP^ only colonies, confirming that high TBXT expression in individual Sox2^mCh^pos;Tbxt^GFP^high cells strongly predisposes them to a mesodermal outcome (Fig 5C). From the population analysis (Fig 3D), we can infer that double-negative descendants of Sox2^mCh^pos;Tbxt^GFP^pos cells are predominantly differentiated descendants of Tbxt^GFP^pos mesoderm. In these clonal assays, double-negative cells were mainly produced by Sox2^mCh^pos;Tbxt^GFP^high cells, consistent with this interpretation. Interestingly, Sox2^mCh^pos;Tbxt^GFP^high cells showed higher plating efficiency than Sox2^mCh^pos;Tbxt^GFP^low cells, suggesting that increased TBXT promotes higher survival and/or adhesion to the substrate (Fig 5C). Thus, at clonal level, high levels of Tbxt^GFP^ predict a high frequency of mesoderm-only outcomes, and an overall increased survival on multiple substrates. Although we have not formally addressed why TBXT high cells display higher survival/colony forming abilities, this might be related to observations in chick embryos where, in a mathematical model based on *in vivo* observations, TBXT high cells in the NMC region display higher motility and the ability to generate higher number of mesodermal cells than TBXT low cells [18].

Substrate composition also seemed to differentially affect cell phenotype. Strikingly, Matrigel and Geltrex reduced the overall mesoderm predisposition of Sox2^mCh^pos;Tbxt^GFP^high cells. This suggests that despite their propensity to differentiate towards mesoderm, Sox2^mCh^pos;Tbxt^GFP^high cells retain a substrate-dependent potential for neuromesodermal differentiation. Intriguingly, although the plating efficiency of both Sox2^mCh^pos;Tbxt^GFP^low and high cells was particularly poor on gelatine, this substrate appeared to enrich for growth of double-positive cells from Sox2^mCh^pos;Tbxt^GFP^low cells (Figs 5C and S3D–S3F). Moreover, the substrate impacted the morphology of the colonies in culture: on gelatine, cells grew as compact colonies and produced an overall higher number of cells per colony, compared to Matrigel culture, where cells formed flat, dispersed colonies (S3E Fig). These observations suggest that once established from Sox2^mCh^pos;Tbxt^GFP^low cells, double-positive cells are favoured by self-adherent growth. Lastly, we found that Sox2^mCh^pos;Tbxt^GFP^high colony survival on Geltrex and Matrigel was comparable, but Matrigel-coating increased the clone survival of Sox2^mCh^pos;Tbxt^GFP^low cells. Thus, Sox2^mCh^pos;Tbxt^GFP^low and high cells exhibit differential behaviour that is modified by the substrate, which can alter both the cell phenotype and the extent of double-positive cell propagation.

We next compared individual colony composition with input reporter level (Fig 5A and 5D). Cells in the Sox2^mCh^pos;Tbxt^GFP^high gate over two experiments predominantly produced only Tbxt^GFP^-positive mesoderm and/or double-negative presumptive mesoderm (Fig 5D, high GFP ‘plateau’ colonies). These two experimental repeats derived from populations where differentiation was relatively advanced and most cells lay in a Tbxt^GFP^ high;Sox2^mCh^ low state just prior to exit from the double positive population (S3A and S3B Fig – Exp 1–2). This suggests double positive cells reach a ‘mesoderm specification’ threshold beyond which mesoderm formation is virtually the only outcome and implying that some Sox2^mCh^; Tbxt^GFP^high cells are not NMPs, recalling our previous observation that, *in vivo*, a minority of SOX2/TBXT immunofluorescent cells are present at the midline, where we detect only mesoderm potential [17,32].

To investigate the relationship between input and output levels of Tbxt^GFP^, we then indexed a proportion of the input population, expanding the ‘Tbxt-high’ gate to include medium-level GFP-expressing cells, to assess whether the clone composition varies as a continuum or shows threshold responses to either reporter. Ordering clones by their input level of Tbxt^GFP^ (Fig 5D) showed an intriguing correlation between fate and input level, whereas using Sox2^mCh^ levels to order the clones showed no discernible correlation (S3C Fig).

We used the indexed cells to mathematically determine whether the clonal lineage of individual NMP progeny is controlled by the initial levels of *Sox2* or *Tbxt* expression and the cell substrate. Sox2^mCh^ and Tbxt^GFP^ expression levels recorded at the time of plating (time = 0), were designated *S* and *T*, respectively. After five days, each seeded cell gave rise to varying numbers of cells (*n*) with four phenotypes based on their expression of TBXT and SOX2: *n*_*S−T+*_, *n*_*S+T−*_, *n*_*S+T+*_ and *n*_*S−T−*_.

To determine whether the clonal lineage at day 5 post-index sort was predominantly mesodermal or neural, we evaluated the difference between numbers of Sox2^mCh^ and Tbxt^GFP^ single-positive cells per colony (*n*_*S−T+*_ − *n*_*S+T−*_). The histogram of *n*_*S-T+*_- *n*_*S+T*-_ determined at time = 5 days, resembled a Gaussian distribution centred at *n*_*S−T+*_ − *n*_*S+T*−_ = zero (S3G Fig).

Of note, many NMPs had small *n*_*S−T+*_ − *n*_*S+T*−_ values (close to zero) and it would be rather arbitrary to identify these NMPs as mesodermal or neural biased. Instead, the tails of the distribution should correspond with high confidence to mesodermal (*n*_*S−T+*_>> *n*_*S+T*−_) or neural (*n*_*S−T+*_ << *n*_*S+T*−_) biased clones. Therefore, we used the standard error of the mean (SEM) of the distribution as a cut-off to assign NMP lineages, even though this excludes colonies from our analyses. Therefore, we classified the lineage of the progeny as mesodermal if *n*_*S−T+*_ > *n*_*S+T*+_ by 27 cells (where 27 corresponds to the SEM). Conversely, we consider the clone to be neural biased when *n*_*S+T*−_ > *n*_*S+T*+_ by 27 cells. To investigate whether the lineage of the NMP progeny at time = 5d depends on the Tbxt^GFP^ or Sox2^mCh^ level of the initial index-sorted NMP cell at time = 0, we plotted the lineage classification of each seeded NMP as a function of both Sox2^mCh^ and Tbxt^GFP^ (S3H and 5E Figs, respectively). To do this, we discretized the scales of Sox2^mCh^ and Tbxt^GFP^ by selecting bins of equal numbers of colonies.

The mesoderm or neural bias of the NMP progeny did not appear to be affected by the level of Sox2^mCh^ in the Sox2^mCh^pos;Tbxt^GFP^pos starting population (S3H Fig). In contrast, the starting level of Tbxt^GFP^ strongly influenced neural/mesodermal bias of the clones (Fig 5D and 5E). At low values of Tbxt^GFP^, most NMPs gave rise to neural-biased clones (Fig 5E). As Tbxt^GFP^ approached a value of 1,745 arbitrary units in the index sort, the proportion of mesoderm-biased versus neural-biased clones rapidly increased (Fig 5D and 5E). Although the ratio of Sox2 to Tbxt has been cited as an important factor determining NMP fate [20,39], this ratio did not predict the clonal outcome better than the levels of Tbxt^GFP^ alone (S4C–S4F Fig). The substrate, however, did appear to modulate differentiation: neural-biased colonies were more abundant on fibronectin and Matrigel, whereas we did not observe a particular bias towards either lineage when cells were plated on gelatine or Geltrex of lineage-biased Sox2^mCh^pos;Tbxt^GFP^pos cells (S3I Fig). These results suggest the existence of a substrate-sensitive Tbxt^GFP^ expression threshold value (‘N-M bias swap threshold’) above which NMP cells are more likely to produce mesodermal cells, in addition to the mesodermal specification threshold described above.

To gain insight into fate decisions in *in vitro*-derived NMPs, we developed a minimal mathematical model of the proliferation and differentiation dynamics of individual NMPs (see Materials and methods for details). We assumed that each NMP cell can divide symmetrically to: (i) self-renew, giving rise to two NMPs (SOX2pos;TBXTpos); (ii) differentiate into two mesoderm cells (SOX2neg;TBXTpos; (iii) differentiate into neurectoderm (SOX2pos; TBXTneg); and (iv) differentiate into double-negative cells (SOX2neg;TBXTneg). Thus, our model has four parameters, which are the rates by which NMP cells give rise to each outcome. To check whether the model is sufficient to reproduce the dependence of the neural/mesodermal decision on the initial Tbxt^GFP^ levels in NMPs, we fitted our model to the experimental data.

When we grouped NMPs according to cell substrate, we observed that gelatine was associated with the highest rates of self-propagation of NMPs and their differentiation towards mesoderm (SOX2neg;TBXTpos), as well as descendants of nascent mesoderm, which are double negative (S4A Fig). Matrigel, on the other hand, produced the highest rate of differentiation towards neuroectoderm (SOX2pos; TBXTneg) (S4A Fig). Classifying NMPs according to whether they had Tbxt^GFP^ levels below or above the Tbxt^GFP^ threshold reported above (regardless of substrate), showed that Tbxt^GFP^ values above the threshold lead to a higher differentiation rate towards mesoderm, whereas Tbxt^GFP^ values below the threshold lead to neuroectoderm bias (Figs 5F and S4E), consistent with our results shown in Fig 5E. Interestingly, for both populations, cells produced more NMPs at higher rates than either neural or mesoderm cells. This suggests that the threshold in differentiation bias does not affect the ability of NMPs to self-propagate and distinguishes this lower ‘N-M bias swap’ threshold from the high-Tbxt^GFP^ ‘specification threshold’, where cells predominantly exit the NMP state towards mesoderm differentiation. Despite the ability of fibronectin and Matrigel to bias cells towards neural differentiation, the mid-level threshold still holds when the experimental data are disaggregated according to the coating used (Fig 4B and 4F).

Thus, clonal analysis and mathematical estimation indicate that individual Sox2^mCh^pos;Tbxt^GFP^pos double-positive cells *in vitro* are NMPs, and that threshold levels of *Tbxt* determine whether they are neural-biased or mesoderm-biased (N-M bias swap threshold). Levels of Tbxt^GFP^ in combination with Sox2^mCh^ determine mesoderm specification (mesoderm specification threshold; summarised in Fig 5G). The cell substrate can modulate the proportions of neural versus mesoderm cells formed, while gelatine, a relatively challenging substrate for NMP adherence, once cells are attached, favours self-adherence and more efficient NMP self-propagation.

### 6. TBXT and SOX2 *in vivo* spatial patterns provide separate information to NM fated regions

Our clonal analysis *in vitro* indicates that TBXT predicts NM fate bias with SOX2 depletion contributing to mesoderm specification. Our result implies that each gene inputs separate information to NMP fate decisions. This contrasts with previous gene regulatory network models of NMP fate regulation, which suggest that NMPs interpret the ratio of SOX2 and TBXT to balance fate choices, with this ratio arising from antagonistic interactions [15,18,20,40]. This model would (at its simplest) suggest that the information provided by SOX2 to a cell is the opposite of that provided by TBXT, and vice versa, meaning that the two inputs can effectively compress into one channel.

**Fig 6:**
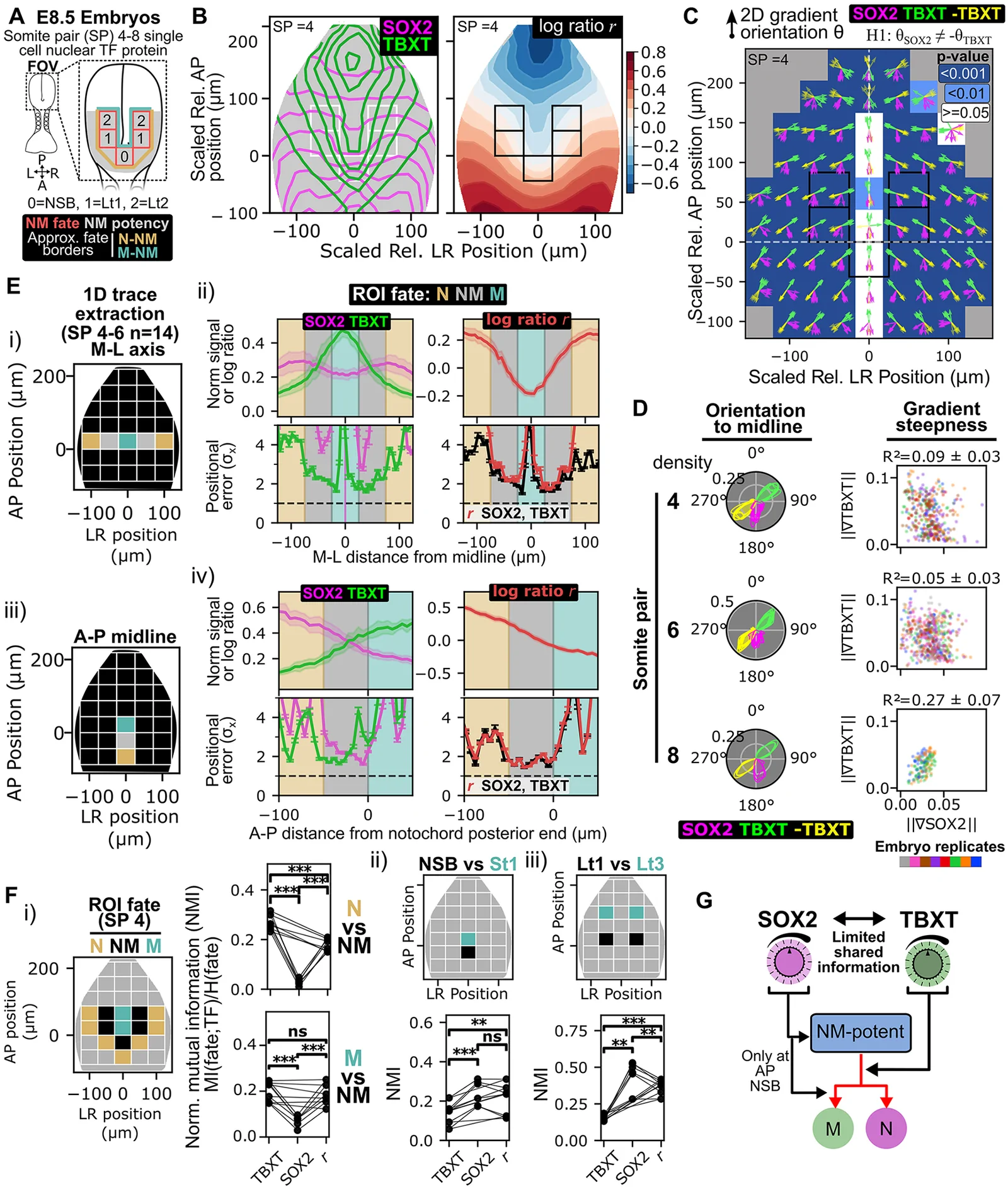
TBXT and SOX2 spatial patterns show high precision and independence. **(A)** Schematic of imaged E8.5 epiblast tissue to collect single cell nuclear signal of TBXT and SOX2. Map of NM fated regions, node streak border = NSB, Lateral 1 = Lt1, Lateral 2 = Lt2, within the NM competent region and approximate N-NM and M-NM fate borders. **(B)** Average spatial contours in normalised 2D space of nuclear SOX2, TBXT, and the log ratio in 4 somite pair (SP, *n* = 6) embryos. Squares indicate NM fated regions. **(C)** 2D gradient orientations of TBXT, SOX2 and the negative of TBXT in 4SP embryos. Statistical significance in the difference between negative TBXT and SOX2 was performed by MANOVA and false discovery rate (FDR) correction. Statistical significance threshold was set at *p* < 0.05. **(D)** Distribution of orientations during subsequent somite pair stages show consistency of relationships observed at 4SP. Comparing gradient steepness between TBXT and SOX2 shows low correlation as calculated by R2 per embryo. **(E)** TBXT and SOX2 gradient precision evaluated in 1D tissue traces extracted in absolute space (microns) through the (i, ii) medial–lateral (M–L) axis just posterior to the NSB or through the (iii, iv) anterior-posterior midline. Both traces include N, NM, and M fated regions. **(F)** Comparison of differences in normalised mutual information (NMI) shared between regions for SOX2, TBXT, or the log ratio (*r*) across (i) whole NM-M or NM-N borders or (ii,iii) comparing subsets of the M vs. NM regions. NMI was calculated at the single-cell level per embryo and paired *t t*ests with *p* < 0.05 thresholds and false discovery rate correction were used for significance. *** = *p* < 0.001, ** *p* < 0.01, * *p* < 0.05. **(G)** Graphic representation of independent toggles where TBXT overall provides high-precision information for both cell fate decisions, and SOX2 mostly provides broad low-precision information that corresponds to NM potent regions. Data for Fig 6B–6F: Data files 1 (https://doi.org/10.5281/zenodo.15802710) and 4 (https://doi.org/10.5281/zenodo.21676159).

This ‘antagonism’ model predicts SOX2 and TBXT would form opposing gradients in the epiblast and the ratio provides enough information for cells to make accurate fate decisions. Alternatively, if the genes provide separate information, expression gradients would not align, i.e., spatial variation in levels of one protein must be unexplainable by the other. A prediction of this ‘separate information’ hypothesis is that if there is unshared variation between two genes, compressing to one input will result in information loss and reduced positional information. To test these hypotheses, we re-analysed our published 3D confocal image dataset of TBXT and SOX2 immunofluorescence in the E8.5 mouse caudal epiblast. These images were computationally flattened to produce 2D projections of anterior–posterior (AP) and medial–lateral (ML) positions (normalised in ML to the epiblast edge and in AP by the primitive streak length, then scaled to average real distances), at somite pair stages (SP) 3–9 (Fig 6A, data from [17]). In these E8.5 mouse embryos, the neural or mesodermal fate of epiblast regions is known in detail [8,32], allowing precise correlation of protein levels with cell fate [17]. We evaluated the extent to which spatial positional information carried by SOX2, TBXT, or the compressed SOX2/TBXT ratio, relates to boundaries between regions of distinct fates and potency.

First, we provide a quantitative interpretation of the antagonism model in the form of a log ratio function between SOX2 and TBXT (*r*) that compresses two signals into one channel. It can be derived as a subtraction of log(SOX2)-log(TBXT), where:


$$\begin{array}{c} \;=\;log(\frac{SOX\text{2}}{TBXT})\;=\;-log(\frac{TBXT}{SOX\text{2}})\;=\;log(SOX\text{2})-\;log(TBXT) \end{array}$$ (1)

The log ratio satisfies the required balancing behaviour by providing symmetry between SOX2 and TBXT variance. In this hypothetical scenario, balanced SOX2/TBXT would indicate a NM state. Increasing *r* from higher SOX2 or lower TBXT values beyond a hypothetical threshold would result in neural (N) fate, and vice versa for mesoderm (M) fate by higher TBXT and lower SOX2.

We previously reported that SOX2 forms a gradient whose contours are mostly linear and parallel to the AP axis, whereas the TBXT gradient is non-linear, forming diagonal V-shape contours changing across both ML and AP axes [17]. This suggests that the antagonism model, which predicts that SOX2 and negative TBXT gradients would align, does not fully explain these observations. To test this, we compared the patterns of TBXT, SOX2, and their log ratio (predicted by the antagonism model), with the borders between NM and either neural or mesodermal fate via 2D projected maps of the epiblast. These show that the log ratio pattern roughly aligns with the NM fate boundaries (Figs 6A, 6B, and S5A–S5D). However, the SOX2 and TBXT expression patterns are much more complex than recapitulated by the log ratio. The difference between SOX2 and negative TBXT gradient orientations was statistically significant in almost all epiblast locations, only aligning at the midline (Fig 6C), indicating the antagonism model cannot explain most of the TBXT/SOX2 pattern. The distribution of mostly AP SOX2 and diagonal TBXT orientations is maintained over at least the ~12 h during which embryos develop from the 3- to 9SP stage (Figs 6D and S5E). Furthermore, the gradient magnitudes of SOX2 and TBXT do not correlate well (Fig 6D). From this, we infer that SOX2 cannot be providing much ML information, and that much of the TBXT pattern complexity is not produced by an antagonistic relationship with SOX2, since if TBXT and SOX2 cross-repression was predominant, we would expect concomitant changes in SOX2 and TBXT expression patterns. However, SOX2 and TBXT gradients do align at the midline suggesting there may be mutual antagonism in this subregion.

To quantify if increased precision of the TBXT and SOX2 gradient patterns corresponds to one or more fate borders, we leveraged established methods previously used to quantify the positional error that morphogen gradients carry [41]. We used this approach to ask whether cells could, in theory, discern neighbouring positions from varying levels of TBXT and SOX2 concentration in space, which would in turn translate into cell-fate decisions. To perform this, in line with previous implementations, we extracted one-dimensional traces in the ML or AP axes that cross N, NM, and M region fate borders (Fig 6Ei and 6Eiii). Each bin in the ML or AP absolute space is calculated as approximate cell widths (5.47-µbins). In the ML axis, where SOX2 and TBXT gradient orientations were not aligned, we observed a steep, non-linear gradient of TBXT and a much shallower SOX2 gradient (Fig 6A and 6Eii). Because the TBXT gradient was much steeper than the SOX2 gradient along this axis, the log ratio mainly recapitulated the TBXT gradient. Furthermore, distinct areas of precision (represented by local minima in error) existed: the lowest error in the SOX2 gradient (~3–4 cell widths) aligned with the NM-M border, while TBXT showed lower levels of error (2–3 cell widths), spanning both NM-M and NM-N borders (Fig 6A and 6Eii). The log ratio showed information loss when compared to TBXT and SOX2 simultaneously: error increased at the NM-N border, suggesting that the ratio assumption does not hold for fate decisions across the ML axis (Fig 6A and 6Eii).

In the AP midline, SOX2 and TBXT formed opposing non-linear gradients, which is recapitulated as a continuous non-linear slope in the log ratio (Fig 6Eiv). Calculating the positional error in terms of cell widths revealed that SOX2 and TBXT gradients had separate peaks in precision across the NM-M and NM-N fate boundaries, respectively (Fig 6Eiv), both with a precision of approximately two cell widths at these locations. This confirms that while SOX2 and TBXT form opposing gradients, the shapes of the gradients indicate separate regions of peak precision. Interestingly, the log ratio is comparable to considering the two genes as independent variables, both with a consistent two-cell-width error across the NM region (Fig 6Eiv). Thus, the gradient precision along the ML axis is inconsistent with the antagonism model’s prediction that the SOX2 and TBXT gradients would approximate the same levels. However, the midline AP axis may indicate some level of antagonism.

From the above analysis, where multiple single-cell values were averaged to represent as a 1D trace (Fig 6Ei and 6ii), it appears that SOX2 and TBXT confer independent information. However, it would be informative to compare in single cells how much information is shared by these proteins across regions of differing fate. We therefore quantified normalised mutual information (NMI) between each gene (TBXT, SOX2, or the log ratio) across binarised N versus NM or M versus NM regions. This showed that in the ML axis, TBXT shared significantly more information with the N versus NM and the M versus NM fate boundaries than SOX2 (Fig 6Fi). Furthermore, the log ratio was a very poor predictor of N versus NM fate, but a slightly better predictor of M versus NM fate. This suggests that, as cells exit the NMP compartment, TBXT levels are at least as predictive as the log ratio, and, for neural fate, much more so, of their eventual N or M fate. Moreover, SOX2 provides relatively little information in these fate decisions.

At the NM-M fate boundary in the node-streak border, however, SOX2 shared significantly more information than TBXT, but not the log ratio (Fig 6Fii), agreeing with our observation that SOX2 has a peak of precision at this border (Fig 6Eiv). A further comparison between an anterior region of the caudal lateral epiblast containing NMPs with mixed N and M fates and a more posterior, predominantly M-fated, region shows that here too, SOX2 shared significantly more information than TBXT or the log ratio (Fig 6Fiii). Thus, information provided by TBXT predominates over SOX2 in the ML axis, but SOX2 provides slightly more information than TBXT in the AP axis. Finally, we assessed our ability to pinpoint a cell’s location within NM regions based on single-cell profiles of TBXT and SOX2, the log ratio, or both TBXT and SOX2 simultaneously. This analysis showed that only considering both genes as independent variables can allow for an accurate “triangulation” of cells to their AP and ML position in the epiblast (S6 Fig).

Together, these results suggest that between TBXT and SOX2, TBXT provides much of the predictive power for cell fate decisions and displays highly precise gradients (Fig 6G). SOX2 provides a shallow, predominantly AP, gradient with no power to independently predict NM to M decisions, except at the NSB. Furthermore, only at the midline do the SOX2/TBXT gradients approximate the ‘antagonism’ model, and even here, they reach maximal precision at different locations. Thus, we conclude that, as in the clonal analysis (Fig 5) SOX2 and TBXT do not predominantly fit a mutually-repressive model, and TBXT levels are generally more predictive of NMP fate decisions than SOX2 ones.

## Discussion

We generated a minimally disruptive embryonic stem cell line, STR-KI, that faithfully reports on *Tbxt* and *Sox2* expression. This cell line mirrors endogenous protein expression and minimises adverse phenotypic effects, lending confidence to the studies of lineage dynamics performed here. Reduction in SOX2 function negatively affects ESC maintenance and differentiation [16], while reduction of *Tbxt* levels disrupts tail length [11] and cell movement of *Tbxt*+/null cells in chimaeras is defective [42]. Therefore, it is likely that normal protein function is preserved in this cell line since no abnormal phenotype or distribution of cells was detected *in vivo* or *in vitro*.

Our study of the differentiating population over time indicates that ESCs during NMP-like differentiation first reduce Sox2^mCh^ expression when exposed to FGF2, as expected in the epiblast *in vivo* [16]. Then, after CHIR addition, the double-positive population (Sox2^mCh^pos;Tbxt^GFP^pos) emerges from these Sox2^mCh^-low cells, mimicking *in vivo* NMC emergence. Over the next 48 hours, Tbxt^GFP^ expression increases, until Sox2^mCh^ expression disappears from Tbxt^GFP^-high cells. Tbxt^GFP^ is later extinguished, resulting in a double-negative population. This cellular trajectory fits well with the known fate of epiblast cells that will eventually become NMPs *in vivo*: distal epiblast cells express relatively low *Sox2* levels and exit pluripotency to enter a bipotent NMP state in the caudal lateral epiblast or node-streak border. If NMPs encounter the primitive streak at the midline they will up-regulate *Tbxt*, undergo epithelial-to-mesenchymal transition and exit the streak and its WNT-high environment as mesoderm, thereafter downregulating *Tbxt* [8,17,43].

*In vivo*, axial elongation is sensitive to perturbation of both WNT and NOTCH signalling, at least partly because NMPs require WNT for maintenance [8] and both WNT and NOTCH for continued mesoderm production [8,36,44]. TBXT is directly activated by WNT signalling [45], and optimal levels of TBXT require NOTCH signalling [36]. Our observation that inhibiting WNT or NOTCH signalling results in a decrease in TBXT is consistent with this published data. We show, however, that gastruloids undergo a particularly rapid slowing of axis elongation, concomitant with the suppression of Tbxt^GFP^ by either inhibitor (~2–3 h), while Sox2^mCh^ levels change only minimally for 10–15 h. This suggests that adequate levels of both WNT and NOTCH are directly required to maintain TBXT expression and axial elongation.

We further show that single *Tbxt/Sox2* co-expressing cells generated *in vitro* from mESCs (1) can differentiate towards SOX2+ neurectoderm and TBXT+ (and subsequently double-negative) mesoderm, and (2) can self-renew in clonal analyses. These are functional attributes used to define *in vivo* NMPs [4] and formally demonstrate that double-positive cells generated using standard protocols are indeed bipotent NMPs. Our mathematical inference of index-sorted founder cells and the fate in their respective colonies shows that higher Tbxt^GFP^ levels predispose cells towards mesoderm differentiation. Interestingly, this seems to occur at two distinct threshold levels of Tbxt^GFP^. Firstly, double-positive cells with high Tbxt^GFP^ and diminishing Sox2^mCh^ fluorescence appear specified for mesoderm differentiation, reminiscent of a fraction of cells at the primitive streak which express high levels of TBXT and still express SOX2 but are not bipotent on transplantation to NM-fated regions [17,32]. These cells are enriched *in vivo* for TBX6, a factor required to commit cells to a mesodermal fate [26]. In support of this idea, TBX6 expression is elevated in TBXT^GFP^-high cells.

A second mid-level threshold of Tbxt^GFP^ appears to switch cells from a neural-biased to a mesoderm-biased state. This is somewhat unexpected because *in vivo* NMCs, even from predominantly mesoderm-fated regions (which express higher TBXT than those in neural-fated regions), can still produce neurectoderm on transplantation to more neural-fated regions [8]. However, our observation that cell substrate can modify the rate of differentiation suggests that the neural/mesodermal outcome of a clone/colony can also be influenced by environmental cues.

Unexpectedly, we did not find evidence that the ratio of Tbxt^GFP^ to Sox2^mCh^ affected the bias-swap threshold. This contrasts with previous studies suggesting that the TBXT:SOX2 ratio or the ratio of WNT activity to SOX2 determines the outcome of differentiation [18,20,39]. This idea originated from the observations that TBXT and SOX2 expression levels appear to read out the predominant fate of the region *in vivo*: cells expressing high levels of TBXT or SOX2 were more likely to form mesoderm or neurectoderm, respectively, while those expressing low levels of both had more balanced fates [8]. Furthermore, cells lacking TBXT and expressing a *Tbxt*-mCherry reporter showed elevated levels of *Sox2* and other neural markers compared to wildtype [20]. This, and the binding of TBXT and SOX2 near a highly-overlapping set of target genes, suggested a model whereby mutual antagonism between TBXT and SOX2 led to a balanced NMP state that could tip towards mesoderm or neural fates as TBXT or SOX2 levels rose and inhibited the expression of each other and their targets. However, *Tbxt* and mesodermal expression in *Sox2* mutant embryos was not tested in these experiments. Moreover, TBXT^−/−^ NMPs in chimaeras do not seem to up-regulate neural genes, nor overproduce neurectoderm [46]. This suggests the increased neural expression in intact TBXT mutants may be indirect. In experiments measuring the SOX2:TBXT ratio, the levels of Sox2 in the NMP compartment are relatively constant [18,39]. In our analyses of SOX2 and TBXT in NMP-containing regions, we found limited evidence supporting an antagonistic relationship between SOX2 and TBXT: their gradients do not align and reach maximal precision at different locations. Furthermore, the TBXT:SOX2 ratio was not as predictive of cell fate as the separate TBXT, and to a lesser extent, SOX2, levels. Interestingly, at the midline, there did seem to be evidence that the two proteins form an opposing anteroposterior gradient and between the node-streak border and primitive streak, where SOX2 levels were most predictive of cell fate. This data suggests that the two proteins behave as independent inputs over most of the caudal lateral epiblast, with TBXT as a predominant predictor, supporting our *in vitro* clonal data. However, local disturbances at the node-streak border and primitive streak midline may diverge from this scenario.

Our data suggest that, against a baseline of relatively uniform, low SOX2, the TBXT:SOX2 ratio might appear to predict neural versus mesodermal outcome, when TBXT levels are a predominant determinant of differentiation bias. Nevertheless, SOX2 may serve an important function in the NMC population. In zebrafish, ectopic expression of SOX2 in NMCs prevents their exit to the mesoderm [47]. Together with the observation that TBX6 activation upon entry to the primitive streak suppresses SOX2 to prevent ectopic neural differentiation in the paraxial mesoderm [26], this suggests SOX2 threshold levels operate at the point of exit of cells from the NMC population. Interestingly, ordering *in vivo* single NMP transcriptomes along a differentiation trajectory shows that peak *Tbxt* mRNA levels occur in double-positive cells, and *Tbx6* mRNA, signifying mesodermal commitment, begins to accumulate only in cells with lower levels of both *Sox2* and *Tbxt* mRNAs [15]. Our observation that during *in vitro* NMP differentiation, TBXT^GFP^ increases while Sox2^mCh^ levels remain constant, then the levels of both reporters drop before cells exit the double positive population is consistent with such a gatekeeper function of SOX2, but not with a continuously variable mutual inhibition of SOX2 and TBXT.

The primacy of TBXT dosage in influencing the outcome of differentiation recalls previous experiments *in vivo* where levels of TBXT dictate cell behaviours: low-TBXT-expressing cells stay in the primitive streak/tail bud, and exit as mesoderm if TBXT levels are increased [42,48]. Our study indicates that cells sense cues in their environment (e.g., WNT, NOTCH), respond by tuning TBXT up or down, which in turn influences the differentiation outcome at the single cell level. Furthermore, quantitative reduction in wildtype TBXT gene dosage progressively shortens the axis [11,49,50]. This suggests that continued axial elongation (through the persistence of NMPs) is sensitive to TBXT levels through modulating these probabilistic states. Since we show that gastruloid manipulation during elongation displays this TBXT sensitivity, such stem-cell-based models will prove useful in the future to investigate the mechanistic bases of these molecular thresholds during developmental processes, such as axis elongation.

## Materials and methods

### Reporter mESC generation

To generate the double reporter cell line, we performed CRISPR-based transgenesis in two targeting rounds (one for each gene locus, starting with *Tbxt*) in E14tg2a ESCs following an established CRISPR-Cas9 protocol [23]. crRNA and sgRNA guides are listed in S1 Table. Briefly, we replaced the stop codon at the C-terminus of the targeted genes by homologous recombination with a reporter construct containing T2A-mCherry (in the case of *Sox2*) or P2A-H2B-GFP (in the case of *Tbxt*) (Fig 1A). The combination of nuclear-localised GFP and whole-cell mCherry aided detection of colocalised expression while minimising potential disruption to normal cellular function. The *Tbxt* targeting vector contained a puromycin resistance cassette, which was used for clone selection. *Sox2*-expressing cells were selected by FACS, sorting individual mCherry+ mESC into 96-well plates. Single clones were picked, expanded, and verified for correct transgene integration by PCR and nanopore sequencing (S1A and S1B Fig).

### PCR and nanopore sequencing

Correct integration of the fluorescent protein sequences into the endogenous loci of *Tbxt* and *Sox2* was confirmed by PCR amplification of the targeted site. The primer sequences used are listed in S1 Table. To validate the sequence, PCR products were subcloned into a plasmid using the Zero Blunt TOPO PCR Cloning Kit and sequenced via Nanopore sequencing.

### Mouse husbandry

C57BL/6 female mice (Charles River) were used for chimaera generation. MF1 mice were used for electroporation experiments. ICR wildtype mice (JAX stock #009122, The Jackson Laboratory) were used for immunostaining. Mice were maintained on a 12-h light/12-h dark cycle. To collect embryos at specific developmental stages, timed matings were set up overnight. Noon on the day of finding a vaginal plug was designated as embryonic day (E) 0.5. Chimera experiments were performed under the UK Home Office project license PEEC9E359 and were approved by the Animal Welfare and Ethical Review Panel of the University of Edinburgh and within the conditions of the Animals (Scientific Procedures) Act 1986. Embryos dissected for immunostaining were approved by the Ethical Committee of the RIKEN Center for Biosystems Dynamics Research (A2016-03-13).

### Chimaera generation

C57BL/6 female mice were superovulated (100 IU/mL PMSG and 100 IU/mL hCG intraperitoneal injections 48 h apart) and crossed with wild-type studs. Pregnant mice were culled at E2.5 by cervical dislocation, ovaries with oviducts were dissected and collected in pre-warmed M2 medium. Oviducts were flushed using PBS and a 20-gauge needle attached to a 1 mL syringe and filled with PB1 [51]. E2.5 embryos were collected and washed in PB1, their zona pellucida removed using acidic Tyrode’s solution and transferred to a plate with incisions where of 8–15 cells were added to each embryo. Embryos were then incubated at 37 °C in 5% CO_2_ for 24 h prior to transfer to pseudopregnant females. Blastocysts were selected and collected to be transferred into the uterus of a pseudopregnant CD-1 female. Females were culled according to Schedule 1 (Animals (Scientific Procedures) Act 1986), and embryos were dissected at E6.5–9.0 in M2 medium and observed for chimeric ESC contribution under an Olympus IX51 microscope. The sex of embryos used in this study was not determined. All reagents are listed in S1 Table.

### Focal electroporation and fate mapping

A more detailed description can be found in [52]. E7.5 neural plate (NP) stage embryos were dissected in M2 medium with their extraembryonic cavities intact, but Reichert’s membrane removed. A PBS-filled 30 mm petri dish was prepared under a stereomicroscope (Zeiss Stemi 2000-C) with a PBS-filled, capillary-sheathed, platinum point electrode (anode; final diameter of capillary sheath 20–30 µm) and an 0.2 mm L-shaped platinum wire (cathode) connected to an ECM 830 square wave pulse generator (BTX). Using a pneumatic pico pump (World Precision Instruments, PV830) and micropulled injection needles, a small volume (<5 µL) of DNA solution (pCAG::GFP plasmid at 1–1.5 μg/mL, 0.01% Fast Green food colouring dye in PBS) was injected in the amniotic cavity of an NP-stage embryo. Embryos were briefly transferred from M2 medium to the PBS-filled electroporation dish, and electroporated using 200 Volts in 6 pulses, each of 50 ms duration with a 1 s interval between each pulse. Embryos were then immediately transferred to pre-equilibrated culture medium and cultured in 4-well plates in an incubator supplied with 5% CO_2_ in air at 37 °C for 24 h [32]. GFP contribution was assessed at 2 h post-electroporation using a fluorescence compound dissecting microscope (Nikon AZ100). For assessment of contribution, samples were fixed, immunostained as wholemount as described below using an anti-GFP primary antibody (Abcam ab13970, 1:800) and Liquid DAB+ Substrate Chromogen System (Dako K3467). Imaging and scoring of contribution were done under a Nikon AZ100 microscope.

### Immunofluorescence on wholemount embryos

E6.5-E8.0 embryos were dissected in home-made M2 medium [53], their yolk sac and amnion membranes removed, after which they were fixed for 25 min at 4 °C in 4% PFA solution (Nacalai Tesque #09154-85). After three short washes in PBS in 0.1% Triton X-100 (now referred to as PBST), samples were permeabilized in 0.5% Triton X-100 in PBS for 15 min, followed by 20 min in 0.5 M glycine in PBST, two 10 min washes in PBST and blocked overnight at 4°C in 10% donkey serum (Merck) in PBS/0.3% TritonX100. Primary antibodies were diluted in blocking buffer for 48 h on a rocking platform at 4 °C. Antibodies used (supplier, catalogue number, final concentration): anti-Sox2 (Abcam, ab92494, 1:200); anti-TBXT (R&D, AF2085, 1:200); anti-TBX6 (R&D, AF4744, 1:1,000). After two days, four 25 min washes were performed with PBST on a rocking platform at room temperature. Alexa Fluor-conjugated secondary antibodies (Thermo Fisher Scientific) were diluted in blocking buffer at 2 µg/ml final concentration and incubated for 48 h at 4 °C, then washed in PBST (4 × 25 min) with the last wash containing DAPI (Invitrogen, D3571, final concentration at 2.5 µg/mL). To image, samples were cleared in BA:BB (2:1 benzyl alcohol:benzyl benzoate; Sigma) after dehydration through an increasing methanol (Nacalai Tesque)/PBS steps (25%, 50%, 75%, 2 × 100%, 5 min each).

Whole-mount embryo samples were imaged under a LSM800 confocal system using GaAsP detectors (Zeiss). Three replicate embryos were imaged per developmental stage. Whole-mount immunostaining data was processed using Zeiss software (Zeiss). For DAPI channel processing, a median filter and background subtraction were applied. Orthogonal projections were generated as *X*–*Y* weighted averages of 8 consecutive slices, representing a total of 8 µm across the Z-axis. S1 Movie was generated using Imaris (Oxford Instruments) from confocal z-stacks of immunostained wild-type embryos. TBXT-positive cells were rendered as filled volumes and SOX2-positive cells as surfaces.

### Cell culture, differentiation and flow cytometry/FACS

STR-KI mESCs were maintained on 0.1% gelatine-coated plates in Glasgow Minimum Essential Medium (GMEM, Sigma-Aldrich, G5154) supplemented with 10% foetal calf serum (Gibco, 10270-106), 100 U/mL LIF (made in-house), 100 µM 2-mercaptoethanol (Gibco, 31350-010), 1× non-essential amino acids (Gibco, 11140-035), 2 mM L-Glutamine (Invitrogen, 25030-024) and 1 mM Sodium Pyruvate (Invitrogen, 11360-039). mESCs were passaged every other day using 1× trypsin (Sigma, 59429C) after washing with PBS.

For NMP differentiation, following [25], STR-KI mESCs were plated at a density of 5,000 cells/cm^2^ on 0.1% gelatine-coated plates in N2B28 medium. N2B27 medium consisted of a 1:1 mixture of Neurobasal (Thermofisher, 21103049) and Advanced DMEM/F12 (Thermofisher, 12634028) supplemented with 2 mM L-Glutamine, 50 µM 2-mercaptoethanol, 0.5× B27 (Thermofisher, 17504001) and 0.5× N2 (Thermofisher, 17502048). Cells were supplemented with 10 ng/mL FGF2 (R&D Systems #3718-FB) for 48 h, and for additional 24 h with both 10 ng/mL FGF2 and 5 µM CHIRON99021 (Axon #1386).

For flow cytometry analyses, after TrypLE Express Enzyme 1× treatment and centrifugation, cells were resuspended in PBS containing 2% FCS and 0.1 µg/mL Draq7. For FACS, resuspension was done in N2B27 medium containing 0.1 µg/mL Draq7.

Cells were analysed using a 4 laser LSR Fortessa (BD) flow cytometer, employing B 530/30-A, Y/G 610/20-A, and R780/60-A laser/filter combinations. For cell sorting, a BD FACS Aria II Cell Sorter was used with B 525/50-A, Y/G 610/20-A, and R780/60-A laser/filter combinations.

For single cell plating experiments, differentiated cells were detached using TrypLE Express Enzyme 1× (Gibco 12604013) and sorted into CellCarrier 96-well plates coated with one of the following substrates: 0.1% gelatine, fibronectin (16.6 µL/mL in PBS; Sigma, F1141), Matrigel (200 µg/mL in PBS; Corning, 354277), or Geltrex (120 µg/mL in Advanced DMEM/F12; Thermofisher, A1413201). Plates were coated and incubated for 1 h at 37 °C prior to cell seeding. Cells were cultured in N2B27 supplemented with 100 U/mL penicillin/streptomycin (Invitrogen, 15140-122), 10 µM ROCK inhibitor Y-27632 (Tocris, 1254/10) and either 10 ng/mL FGF2 or in combination with 5µM CHIRON99021. After 24 h, the medium was replaced without ROCK inhibitor. The ROCK inhibitor Y-27632 was used to enhance single-cell survival [54] and was used in FACS experiments to counteract dissociation and cell stress-induced apoptosis.

All cells were maintained under standard culture condition (37 °C and 5% CO_2_).

### Quantitative reverse transcription polymerase chain reaction (qRT-PCR)

A total of 200 cells were sorted into PCR tubes containing 10 µL of 2× Reaction Mix from the CellsDirect One-Step qRT-PCR Kit (Invitrogen, 11753100) with 0.4 U/µl of RNAse inhibitor (Invitrogen, AM2694) and flash-frozen on dry ice. Reverse transcription reactions were performed using the SuperScript III RT/Platinum Taq mix provided in the same kit. qPCR was performed using LightCycler 480 SYBR Green I Master (Roche, 4887352001) in 384-well LightCycler 480 Multiwell Plates (Roche, 04729749001) and using the Roche LightCycler 480 Real-Time PCR System. The *Tbp* gene was used as an endogenous control, and relative gene expression was calculated using the 2^−ΔΔCt^ method.

### Bulk-RNA sequencing

20,000–100,000 cells were sorted into 1.5 mL Eppendorf tubes and total RNA was extracted and preserved using the Zymo Research RNA Shield kit according to the manufacturer’s instructions. Samples were submitted for RNA sequencing to Plasmidsaurus, using Illumina sequencing technology with custom analysis and annotation. Counts per million tables provided in supplementary information were used for downstream analysis. Marker gene lists were manually curated by cross-checking expression in a mouse single-cell RNA-seq atlas [30] for early-expressing genes (NMP to 48h). For a matured neural or mesodermal character of 7d samples, we manually curated the top 50 DEGs between 7d populations and their progenitors through searching E9.5–10.75 images and their annotations in the Mouse Genome Informatics database [31].

### Immunocytochemistry and confocal microscopy of cells

Cells were fixed with 4% paraformaldehyde for 10 min, then washed twice with PBS over a total of 10 min and permeabilised with 0.5% Triton X-100 in PBS for 10 min. Following two additional PBS washes, cells were blocked for 30 min in a solution containing 5% donkey serum and 0.1% Triton X-100 in PBS. Primary antibodies, diluted in the same blocking solution, were added overnight at 5 °C. After washing twice with PBS, secondary antibodies, diluted in blocking solution, were added for 2 h at room temperature. After two further PBS washes, DAPI (Invitrogen, D3571, final concentration at 2.5 µg/mL) was added for 5 min at room temperature, followed by two PBS washes. Imaging of multi-well plates was performed using the High-Content Imaging Opera Phenix Plus from the IRR Imaging Core Facility. Single-cell colony lineage scoring was performed using Signals Image Artist software (Revvity), using the 3D Analysis stack processing pipeline. Image stacks were processed to identify and segment colonies. For cell counting, the Find Nuclei – Method C function was utilised. Following segmentation, both cell number and fluorescence intensity data were extracted from the analysed image sets.

### Intracellular staining analysis for flow cytometry

Cells were dissociated with 1× TrypLE for 3 min and collected by centrifugation. The cells were washed twice in 1× PBS without Mg^2+^ and Ca^2+^, fixed in 4% formaldehyde solution at 37 °C for 10–15 min and subsequently washed in 1× PBS to facilitate intracellular immunostaining. Rabbit anti-SOX2 (Abcam, ab92494) and Goat anti-TBXT (Biotechne, af2085) primary antibodies were diluted in FACS permeabilization solution (1× PBS + 0.1% TritonX-100) at a concentration of 1:200 and added to cells for 30 min. The cells were then washed twice with permeabilization solution before adding secondary antibodies Donkey anti-rabbit-405 and Donkey anti-goat-647 (Invitrogen, a48258 and a21447; respectively) in the same permeabilization solution at a concentration of 1:1,000. Cells were washed again in permeabilization solution twice more before being resuspended in FACS buffer (1× PBS + 2% FCS). FACS was performed on an Attune CytPix Flow Cytometer, and the data analysed using FlowJo and Rstudio.

### Gastruloid culture, live imaging and quantitative analysis

Gastruloid culture was carried out as previously described ([55] and Vianello and colleagues, 2020 [https://doi.org/10.17504/protocols.io.9j5h4q6]). In summary, 2i/LIF/FCS mESCs were washed in PBS before adding 0.05% Trypsin EDTA solution. After cell detachment, 5–10 volumes of fresh 2i/LIF/FCS were added to quench the Trypsin EDTA solution. Cells were transferred to a universal tube and pelleted by centrifugation at 300*g* for 3 min. The media was aspirated, and the pellet was resuspended in cold PBS to make a single cell suspension. A PBS wash was repeated once more, then the cell pellet was resuspended in prewarmed N2B27 to a single-cell solution. Cells were counted and diluted to a solution of 8,250 cells/mL in N2B27 medium, after which 40 μL of this medium (containing ~330 cells) was plated into untreated u-bottom 96 wells and incubated for 48 h at 37 °C and 5% CO_2_. At 48 h, an additional 150 μL of N2B27 and 3 µM CHIR99021 were added to the well. The last half of the additional media was expelled forcefully to dislodge the aggregate but without spilling the medium. The aggregates were cultured for a further 24 h to d3, after which 150 μL of media was removed and replaced with 150 μL fresh N2B27. At d4, gastruloids were washed in N2B27 and then transferred to flat-bottom and black-walled 96-well plates (PerkinElmer) containing ice-cold phenol-red-free N2B27 (Thermofisher, 21041025, 12348017) + 10% matrigel supplemented with LY 411575 (150 nM), IWP-2 (2.5 μM), or an equal amount of DMSO. Gastruloids were left for 5 min to drop to the bottom of the well and then placed into an incubation chamber at 37 °C and 5% CO_2_ to set the Matrigel. After this period, gastruloids were imaged on a confocal microscope (Opera Phenix Plus) every hour for 24 h. Transmitted light and fluorescence signal was collected using epifluorescence and a 20× objective. Excitations of 488 nm and 561 nm, emissions of 522 nm and 599 nm, and exposure times of 0.1 and 0.2 s were used for GFP and mCherry, respectively. XYZ voxel dimensions were 0.89 × 0.89 × 5 microns.

Image analysis was performed in python using custom scripts and open-source libraries that can be found at https://doi.org/10.5281/zenodo.17192095. Whole tissue segmentation masks were generated using with the Segment Anything Model (SAM) (arXiv:2304.02643). Extending poles of gastruloids were inferred by median projecting Tbxt^GFP^ signal z-stacks to 2D, smoothing with a Gaussian filter, and isolating the largest domain of pixels over the 99th percentile. The vector between the centroid of this domain and the centroid of the whole gastruloid mask determines the orientation to the *Sox2/Tbxt* co-expressing region. The orientation calculated at 10 h post-embedding was applied to all other timepoints. Traces were collected in a 90 μm-wide linear line orthogonal to the vector orientation. Extending pole “ends” containing Sox2/Tbxt co-expressing domains were isolated as a bin between 35 and 178 μm from the end of the pole. The limits of this region were manually determined by the overlap of Tbxt^GFP^/Sox2^mCh^ signal gradients and previous observations of the location of NMP regions *in vivo* (i.e., the tail tip) [17]. Signal of Tbxt^GFP^ and Sox2^mCh^ was measured in 2D by the average intensity from median z-projections of both channels in 0.89 μm-wide bins along the gastruloid trace. Channel signal was normalised per gastruloid to the 5th and 99th percentile of trace signal values at 0 h, then per time point and condition normalised to the 99th percentile of DMSO control. For the NMP region Tbxt/Sox2 dynamics, the difference between signal from treated and control conditions was calculated per replicate and time point.

For flow cytometry analyses, an average of 60 gastruloids were pooled per experiment and bulk analysed at 72 hpa and 30 gastruloids at 96 hpa. Analyses were done using the NovoCyte Penteon analyser (Agilent). Flow data was analysed in R using the CytoExploreR plugin (Dillon Hammill (2021), CytoExploreR: Interactive Analysis of Cytometry Data. R package version 1.1.0. https://github.com/DillonHammill/CytoExploreR).

For imaging, gastruloids were fixed with 4% PFA for 40 min at room temperature and stained with DAPI overnight. Gastruloids were mounted in low-melting-point agarose in a 96-well Ibidi plate and clarified with RapiClear 1.49 overnight. An average of 3–5 gastruloids per condition were imaged using an OperaPhenix confocal microscope, a 300 µm section was imaged in 2 µm steps and representative examples were selected. Image analysis was performed in Fiji. E14tg2a gastruloids were used as a negative control to set fluorescence thresholds. A maximum intensity projection of the DAPI signal was used to create outlines of the gastruloids using a custom Fiji macro.

### Mathematical inference for *in vitro* data

In this study, we propose a minimal mathematical model of the dynamics of NMP cells and their progeny to investigate the cell fate decision process. We assume that each NMP cell divides symmetrically, resulting in (i) two NMPs, or (ii) two cells that are simultaneously Tbxt+ and Sox2−, or (iii) two cells that are simultaneously Tbxt- and Sox2+, or (iv) two cells that are simultaneously Tbxt- and Sox2−. We also assume that, except for NMPs, the other cells do not divide within the time window of our study. The model is encoded in the following linear system of ordinary differential equations describing the time course of NMPs and their progeny:


$$\begin{array}{c} \frac{dNMP}{dt}=\alpha *NMP-\beta *NMP-\gamma *NMP-\delta *NMP \end{array}$$ (2)

$$\begin{array}{c} \frac{dn_{S+T-}}{dt}=\text{2}*\beta *NMP \end{array}$$ (3)

$$\begin{array}{c} \frac{dn_{S-T+}}{dt}=\text{2}*\gamma *NMP \end{array}$$ (4)

$$\begin{array}{c} \frac{dn_{S-T-}}{dt}=\text{2}*\delta *NMP \end{array}$$ (5)

where *t* is time, *NMP*, $n_{S+T-}$, $n_{S-T+}$ and $n_{S-T-}$ are the number of NMPs, the number of cells that are simultaneously Sox2+ and Tbxt−, the number of cells that are simultaneously Sox2− and Tbxt+ and the number of cells that are simultaneously Sox2− and Tbxt−, respectively. The model has 4 parameters: α, β, γ and δ, which correspond to the rates at which NMP cells give rise to more NMP cells, cells positive for Sox2 (S) but negative for Tbxt (T) (i.e., neural lineage), cells negative for Sox2 but positive for Tbxt (i.e., mesodermal lineage) and cells negative for both Sox2 and Tbxt (double negative).

To test whether the model was sufficient to reproduce the dependence of the neural/mesodermal decision on the *Tbxt* level of the NMP, we fitted the model to the experimental data.

This system has an analytical solution given by


$$\begin{array}{c} NMP(t)={NMP}_{\text{0}}*e^{(\alpha -\beta -\gamma -\delta )t} \end{array}$$ (6)

$$\begin{array}{c} n_{S+T-}(t)=\frac{\text{2}*\beta *{NMP}_{\text{0}}*e^{(\alpha -\beta -\gamma -\delta )t}}{\left(\alpha -\beta -\gamma -\delta \right)}+{n_{S+T-}}_{\text{0}} \end{array}$$ (7)

$$\begin{array}{c} n_{S-T+}(t)=\frac{\text{2}*\gamma *NMP_{\text{0}}*e^{(\alpha -\beta -\gamma -\delta )t}}{\left(\alpha -\beta -\gamma -\delta \right)}+{n_{S-T+}}_{\text{0}} \end{array}$$ (8)

$$\begin{array}{c} n_{S-T-}(t)=\frac{\text{2}*\delta *NMP_{\text{0}}*e^{(\alpha -\beta -\gamma -\delta )t}}{\left(\alpha -\beta -\gamma -\delta \right)}+{n_{S-T-}}_{\text{0}} \end{array}$$ (9)

where *NMP*_*0*_ is the initial number of NMP cells, ${n_{S+T-}}_{\text{0}}$ is the initial number of Sox2 positive but Tbxt negative cells, ${n_{S-T+}}_{\text{0}}$ is the initial number of Sox2 negative but Tbxt positive cells and ${n_{S-T-}}_{\text{0}}$ is the initial number of double negative cells.

Finally, we assume the following initial condition, reflecting the experiments described in section 3.


$$\begin{array}{c} NMP_{\text{0}}=\text{1} \end{array}$$ (10)

$$\begin{array}{c} {n_{S+T-}}_{\text{0}}=\text{0} \end{array}$$ (11)

$$\begin{array}{c} {n_{S-T+}}_{\text{0}}=\text{0} \end{array}$$ (12)

$$\begin{array}{c} {n_{S-T-}}_{\text{0}}=\text{0} \end{array}$$ (13)

To fit the model to the experimental data, we minimised the distance function δ between the experimental number of cells and the model-predicted number of all cell types for each experiment. The distance d was defined as:


$$\begin{array}{c} \delta =\sqrt{{\left({NMP}_{e}-{NMP}_{t}\right)}^{\text{2}}+{\left({n_{S+T-}}_{e}-{n_{S+T-}}_{t}\right)}^{\text{2}}+{\left({n_{S-T+}}_{e}-{n_{S-T+}}_{t}\right)}^{\text{2}}+{\left({n_{S-T-}}_{e}-{n_{S-T-}}_{t}\right)}^{\text{2}}} \end{array}$$ (14)

where *NMP*_*e*_ and *NMP*_*t*_ are the experimental and model-predicted number of NMP cells, ${n_{S+T-}}_{e}$ and ${n_{S+T-}}_{t}$ are the experimental and model-predicted number of cells positive for SOX2 but negative for TBXT, ${n_{S-T+}}_{e}$ and ${n_{S-T+}}_{t}$ are the experimental and model-predicted number of cells positive for T but negative for SOX2, and finally ${n_{S-T-}}_{e}$ and ${n_{S-T-}}_{t}$ are the experimental and model-predicted number of cells negative for both SOX2 and TBXT. From all best-fitting parameter values that minimised *d* for each experiment, we calculated the mean and standard error of the mean.

A Jupyter Notebook (http://jupyter.org/) containing the source code used to generate the mathematical inference plots in Figs 5, S3, and S4 can be found at https://doi.org/10.5281/zenodo.17154933.

### Spatial gene expression pattern analysis for *in vivo* data

Analysis and data visualisation was performed using custom Python scripts, open source libraries and published scripts [56–60]. The log SOX2 ratio was calculated by subtracting the log of normalised nuclear signal as per equation (1) (normalisation carried out previously [17]). Gene expression gradients were calculated as a vector of a gene differential in AP and ML directions, where the orientation was calculated as the angle of the vector and steepness as the vector magnitude. Differences in orientations were tested with a MANOVA test, as previously described [61], with Benjamini-Hochberg false discovery rate correction and significance threshold set at 0.05. Orientation to the midline distributions were calculated by inverting the gradients on the right half of the embryo and estimating density with a Gaussian kernel density estimator. Gradient magnitude correlation was calculated using R-squared per embryo to generate mean and standard deviation parameters. Positional gradient error $\sigma_{x}\;$ was calculated using a Python implementation of published code [62] with the equation from [41] below.


$$\begin{array}{c} \frac{\text{1}}{\sigma_{x}(X)}=\left|\frac{d{\overset{―}{g}}_{i(x)}}{dx}\right|\frac{\text{1}}{\sigma_{x}(X)} \end{array}$$

where $\frac{d{\overset{―}{g}}_{i(x)}}{dx}$ is the derivative between x bins in either AP or ML directions. This extends to consider *n* genes by the generalisation:


$$\begin{array}{c} \frac{\text{1}}{{\sigma^{\text{2}}}_{nx}}=\sum_{ij=\text{1}}^{n}\left[\frac{d{\overset{―}{g}}_{i(x)}}{dx}(C^{-\text{1}})\frac{d{\overset{―}{g}}_{i(x)}}{dx}\right] \end{array}$$

Here, C is the covariance matrix of the joint expression profiles of *n* genes, while $C^{-\text{1}}$ denotes the corresponding inverse covariance matrix. When considering a single gene (*n* = 1), this simplifies to *σᵢ*, the standard deviation associated with the expression level of gene gᵢ. Bin widths for 1D traces were set at 5.47 micrometres and approximates one epithelial cell width across the apical-basal plane. Bin width was calculated as the average distance between the four closest neighbours in raw XYZ space. 1D traces were extracted from 50-micrometer-wide lengths in either the M-L plane for A-P traces, and A-P for the M-L plane. Standard error mean bars were estimated using leave-one-out bootstrap with 10 cycles. Mutual information between discretised regions was calculated per embryo as a binary classification between two regions and a single-cell gene as a continuous predictor and 10 neighbours and was normalised to the Shannon entropy [63] of the binary output. Mutual information differences between gene predictors per embryo were tested with a paired *t* test corrected with a false discovery rate and significance threshold of 0.05. Analysis was performed in python using custom scripts and open-source libraries that can be found at https://doi.org/10.5281/zenodo.17192095.

## Supporting information

S1 Movie Node streak border 3D detailed reconstruction of LSEB-NP and LNP stages stained for SOX2 and TBXT. TBXT+ and SOX2+ cells are shown as a filled or hollow surfaces, respectively. Blue circled area in shows TBXT+ cells in the dorsal layer of the node-streak-border (NSB). A, anterior, P, posterior, Ds, distal, Px, proximal. LSEB, late streak early bud; (L)NP, (late) neural plate. PS, primitive streak.
(MP4)

S2 Movie Example of time lapse imaging of a gastruloid following DMSO treatment (bright field). (MOV)

S3 Movie Example of time lapse imaging of a gastruloid following DMSO treatment (Sox2^mCh^ in magenta;Tbxt^GFP^ in green). (MOV)

S4 Movie Example of time lapse imaging of a gastruloid following IWP2 treatment (bright field). (MOV)

S5 Movie Example of time lapse imaging of a gastruloid following IWP2 treatment (Sox2^mCh^ in magenta;Tbxt^GFP^ in green). (MOV)

S6 Movie Example of time lapse imaging of a gastruloid following LY411575 treatment (bright field). (MOV)

S7 Movie Example of time lapse imaging of a gastruloid following LY411575 treatment (Sox2^mCh^ in magenta;Tbxt^GFP^ in green). (MOV)

S1 Table List of reagents used. (XLSX)

S1 Fig STR-KI cell line contributes highly to chimaeras. **(A)** Schematics of targeted regions of the Tbxt and Sox2 loci in the STR-KI cell line showing correct integration and sequence alignment to the consensus after nanopore sequencing. **(B)** Intracellular flow cytometry of day 3 NMPs from STR-KI. (Top) Sox2-mCherry versus Tbxt-GFP dot plot showing the heterogeneous composition of the assayed population. (Middle and Bottom) Flow plots used for the correlation analysis between reporter fluorescence and endogenous SOX2 and TBXT protein levels, respectively, validating reporter fidelity. Representative plots of three independent experiments. **(C)** mouse pre-streak (top) and E8.5 (2–12) somite pair stage (bottom) chimaeras appear morphologically normal, show high contribution of STR-KI cells, and correct location of the reporter fluorescent proteins. Asterisk in D shows a non-contributing littermate as a negative control. Scale bar for pre-streak = 100 mm, for E8.5 = 200 mm. Data for S1B Fig: https://doi.org/10.5281/zenodo.21708690.
(TIFF)

S2 Fig *In vitro*-derived *Sox2*-mCherry and *Tbxt*-GFP co-expressing cells are NMPs, and their lineage trajectory can be captured for potency studies. **(A, B)** qRT-PCR analysis of populations on day 2 and day 4 using the same gating strategy and differentiation protocol in Fig 3A. Gene markers of pluripotency, axial progenitors (A), and neural, mesodermal, endodermal differentiation markers (B) with *n* = 3 (day 2) and *n* = 5 (day 4) independent experiments for each population. **(C)** Repeats of the time-course experiment described in Fig 2C and 2D. A factor of variability between these three experiments can be noted and is inherent to any differentiation protocol. However, cell populations of all three experiments followed the same trajectory trends as summarised in Fig 3D. A representative purity check of the initial FACS populations is shown. **(D)** Experimental schematic for LPM/endothelial differentiation of sorted NMC subpopulations at d3 and sort purity checks for each population. Representative plots from 3 independent experiments. **(E)** Colony examples stained for the LPM marker FLK-1 (white), TBXT (green) and SOX2 (magenta). FLK-1 cells are generated from Tbxt^GFP^-negative or low but are absent from high Tbxt^GFP^-expressing populations. Scale bars = 50 µm. **(F)** Experimental setup for the generation of gastruloids to test symmetry breaking and Tbxt^GFP^ activation upon CHIR treatment. Flow cytometry on an average of 60 gastruloids were pooled and bulk analysed for 72 hpa, and 30 gastruloids for 96 hpa (*n* = 1). **(G)** Immunostaining of wildtype (WT) or STR-KI-derived gastruloids for Sox2^mCh^ and Tbxt^GFP^ at 72 and 96 hpa. Note the absence of Tbxt^GFP^ in both WT and no CHIR-treated gastruloids. Axis elongation is visible between 96 and 120 hpa, depending on the cell line use, with our STR-KI reporter showing elongation from 96 hpa upon CHIR treatment. representative image of 3–5 gastruloids analysed per sample. Data for Fig S2A–S2D, S2F: https://doi.org/10.5281/zenodo.21708690.
(TIFF)

S3 Fig Quantitative analysis and mathematical inference of clonal data. **(A)** Overlap of bulk-flow data from NMP differentiations (shown in Fig 5) where single cells that fell in the Sox2^mCh^pos/Tbxt^GFP^high sorting gate were reanalysed by level of Sox2^mCh^. Exp 1,2 were unindexed, and Exp 4,5 were indexed. The % of cells above the 50th percentile of mCherry were calculated based on the least advanced differentiation repeat (exp5). **(B)** Summary table listing the % of cells within the Sox2^mCh^/Tbxt^GFP^ high population falling in the top 50% percentile of Sox2^mCh^ across all experiments. **(C)** Overview of all individual colonies scored on their percent lineage output, ordered by Sox2^mCh^ level at the time of FACS (day 3). Tbxt^GFP^ levels are also annotated (trace below clonal composition chart). The total number of cells (lower trace) per colony ranged from 2 to 5,335 cells. **(D)** Total number of cells per colony measured after clonal expansion of single cells grown on different extracellular matrices or classified by colony phenotype **(E)**. **(F)** Total number of SOX2+TBXT+ cells per colony cultured on different coating matrices. **(G)** Histogram for *n*_*S−T*+_ − *n*_*S+T−*_ (orange bars). Blue line: Gaussian function fitted to the data. **(H)** Clonally plated Sox2^mCh^ Tbxt^GFP^ (NMP) lineage output as a function of Sox2^mCh^ levels in individually plated NMPs. Each dot represents one colony. The Sox2^mCh^ level on the X-axis is the middle value in the bin. **(I)** Clonally plated Sox2^mCh^/ Tbxt^GFP^ (NMP) lineage output as a function of the extracellular coating used. For C–E: Dots are the mean with error bars, the standard error of the mean. Asterisks indicate statistically significant differences (*p* < 0.05) obtained after applying a Mann–Whitney statistical test (* *p* < 0.05, ** *p* < 0.01, *** *p* < 0.001, **** *p* < 0.0001). Data for S3C–S3F Fig: https://doi.org/10.5281/zenodo.21708690; Data for S3G and S3H: https://doi.org/10.5281/zenodo.21650680.
(TIFF)

S4 Fig Quantitative analysis and mathematical inference of clonal data using the Tbxt absolute levels or Tbxt/Sox2 levels ratio. **(A)** Lineage differentiation rates estimated with our mathematical inference (Tbxt absolute levels), coloured by coating: matrigel (dark green), Geltrex (light green), gelatine (pink) and fibronectin (fuchsia). Dots are the mean with error bars, the standard error of the mean. **(B)** Differentiation lineage rates estimated mathematically (Tbxt absolute levels) sorted by coating used. The initial NMP cells were above (in red) or below (in blue) a Tbxt^GFP^ level threshold of 1,745 arbitrary units (au). **(C)** Colony lineage output when plotting Tbxt/Sox2 ratio over Tbxt absolute numbers. GFP/mCherry threshold value = 0.97. **(D)** Individual colony lineage output as a function of initial Tbxt^GFP^/Sox2^mCh^ ratio in the founder NMP. Each dot represents one colony/experiment, with substrates used: matrigel (dark green), Geltrex (light green), gelatine (pink) and fibronectin (fuchsia). The data was segregated in bins of equal number of colonies and Tbxt^GFP^/Sox2^mCh^ ratio on the horizontal axis is the average per bin. The black line shows a step-function fit to the data, indicating a neural-to-mesodermal lineage transition at ~0.97 Tbxt^GFP^/Sox2^mCh^ [AU]. **(E)** Mathematical estimation of fate in index-sorted NMPs. The rate indicates whether single founder NMPs were above (red) or below (blue) the initial Tbxt^GFP^/Sox2^mCh^ threshold ratio (0.97), as a function of their colony output at the end of the experiment. The dots and the error bars correspond to the mean and the standard error of the mean, respectively. Asterisks indicate significant differences obtained after applying a Mann–Whitney statistical test (*p* < 0.05). **(F)** Differentiation rates of individual lineages estimated mathematically sorted by coating used. The initial NMP cells were above (in red) or below (in blue) the Tbxt^GFP^/Sox2^mCh^ threshold ratio. Dots are the mean with error bars, the standard error of the mean. Asterisks indicate statistically significant differences (*p* < 0.05) obtained after applying a Mann-Whitney statistical test. Data for S4 Fig: https://doi.org/10.5281/zenodo.21650680.
(TIFF)

S5 Fig Log ratio and SOX2, TBXT during E8.5 somite pair stages 3–9. Full data representation across somite pair (SP) stage 3–4,5–7, and 8–9 (labelled 4,6 and 8, respectively). Including **(A)** pairwise density heatmaps of normalised and **(B)** log ratio-transformed single-cell SOX2, TBXT values. Each **(C)** log ratio or **(D)** normalised is mapped in the projected epiblast as 2D contour plots in normalised 2D tissue space. **(E)** In 2D normalised tissue space, the gradient orientations of TBXT and SOX2 where the difference in SOX2 and the negative of SOX2 were statistically tested using MANOVA and false discovery rate correction (FDR). Statistical significance threshold was set at *p* < 0.05. Boxes show NM fated regions in 2D maps. Data for S5A–S5E Fig: Data file 1, https://doi.org/10.5281/zenodo.15802710.
(TIFF)

S6 Fig Comparing the precision of NMC regions TBXT/SOX2 profiles. **(A)** Graphic explaining methodology, where cells in NM fated regions (Node streak border-NSB, Lateral 1, Lateral 2) were isolated and the mean and standard deviation (SD) of SOX2, TBXT, or the log ratio was calculated. Then, cells within one SD of the mean of each gene or a combination of both genes were isolated from the whole epiblast population. The spatial distributions are shown in the projected epiblast as coloured density bins. Accuracy was determined per embryo by the percentage of cells found being either within the original NM region, or any NM region. Only considering both SOX2 and TBXT achieved relatively high accuracy. **(B)** Grid of comparisons between the variables considered (SOX2, TBXT, log ratio, or SOX2 and TBXT) and the region used to define the search distribution (NSB, Lateral1, Lateral 2). Showing how only considering SOX2 and TBXT allows for relatively accurate positioning to the correct region in the epiblast. ± error denotes one standard deviation. NMC = neuromesodermal competent. NM = neuromesodermal (competent region). Data for S6B Fig: Data file 1, https://doi.org/10.5281/zenodo.15802710.
(TIFF)

## Abbreviations

AP: anteroposterior
DEGs: differentially expressed genes
DN: double negative
FDR: false discovery rate
hpa: hours post-aggregation
LNP: late neural plate
LPM: lateral plate mesoderm
NMCs: neuromesodermal competent cells
NMI: normalised mutual information
NMPs: neuromesodermal progenitors
PCA: principal component analysis
qRT-PCR: quantitative reverse transcription polymerase chain reaction
SEM: standard error of the mean
STR-KI: SOX2-TBXT Reporter Knock-In
TF: transcription factor
