# Supplementary material for: Brachyury expression levels predict lineage potential and axis-forming ability of in vitro-derived neuromesodermal progenitors
Source: PLoS Biol. 2026 Aug 25;24(8):e3003960. doi: 10.1371/journal.pbio.3003960 (PMC13533433; doi:10.1371/journal.pbio.3003960)
Supplement: Supplementary file 2 [file pbio.3003960.s016.docx]

**Manuscript number:** RC-2025-03238

**Corresponding author(s): Anahí Binagui-Casas and Valerie Wilson**

## 1. General Statements [optional]

Please see cover letter

## 2. Description of the planned revisions

For clarity, we have grouped our responses to reviewer comments by topic.

***Validation of reporter-endogenous protein***

*Reviewer 1 Minor points:*

*“I wonder whether, Tbxt low cells actually express TBXT protein or not. It might be the case that only a fraction of Tbxt low cells actually express TBXT protein, or none. It would enough to sort the populations showed in Figure 3A and perform immunostaining for endogenous SOX2 and TBXT. This could reveal even better correlations between their levels and cell behaviour”*

*Reviewer 2:*

*Could the authors please comment on the design of the reporter system in a bit more detail? For example, please clarify the necessity to generate a TBXT reporter that includes a H2B-GFP, unlike Sox2, which does not include a H2B. Can the authors distinguish between an increase in the threshold of TBXT, versus an increase detected due to the stability of the H2B-GFP? The low versus high TBXT cells may reflect early versus late TBXT expressing cells. Are the changes in TBXT expression (eg Supplementary Figure 2) significantly changed between the low versus high GFP populations? Additionally, why is the level of GFP similar across low and high GFP populations in Sup Fig 2? Could the in vivo data be used to quantify differences in TBXT (similar to what has been shown previously in Ivanovitch et al., 2021 PMID: 33999917)?*

Our validation has shown a good correlation between TBXT^GFP^ and endogenous protein. In embryos, GFP is correctly localized in the nascent mesoderm (Figure 1B, S1D), while more mature mesoderm anterior to the primitive streak and tail bud does not show GFP expression. Furthermore, gene expression analyses, albeit in bulk, also show a correlation between low and high Tbxt-GFP protein and endogenous Tbxt gene expression. Most strikingly, in figure 1C, endogenous Sox2 and Tbxt correlate closely with their respective reporters. We have now added the R^2^ of our quantifications to the text (line 133). Importantly, our experiments in gastruloids show that, despite the expected stability of the H2B-GFP protein, we can see a decrease in Tbxt-GFP within 3 hours post *Tbxt-*gene downregulation upon inhibitory treatment (Fig 4E), which suggest that GFP fluorescence does not persist when its inductive signals are reduced. Nevertheless, to further clarify how the level of both proteins correlates at the single cell level, **we will perform intracellular flow cytometry and analyse the populations showed in Fig3A, as suggested by R1**. We think that this experiment will provide an excellent validation of our reporter in our main populations of interest (NMP-double positive populations) instead of relying on mRNA levels only.

We will also address the quantification of endogenous TBXT across different NMP regions in vivo as described in the subsection below (*Validation in vivo*).

***Validation of generated derivatives:***

*Reviewer 2:*

1. *Figure 3B - The SOX2+/TBXT- population only shows a moderate level of SOX1 by RT-qPCR. Using pre-neural markers (e.g. NKX1-2) might be helpful here to show progression towards a neural progenitor identity.*
2. *With respect to the mESC differentiation into NMPs (using an established protocol from Gouti et al 2014), after Day 3, cells are cultured in conditions where they are exposed to both CHIR and FGF for a further two days. As these extended CHIR/FGF conditions don't appear to be characterised by Gouti et al., 2014, what proportions and progenitors are generated in the dish under these conditions? The loss of SOX2 by day 5 suggests mesodermal progenitors are the main derivatives but further characterization (eg Meox1/Msgn1) would be needed to verify this claim.*
3. *Further validation of the generated derivatives would also be useful in the re-plating experiments (Figure 3) to test whether the double negative cells are transitioning to a mesodermal (eg Meox1/Msgn1) or neural derivative (eg Sox1/Pax6). Similarly, at day 5 and 6 of the differentiation, there appears to be a loss of Sox2 expression in some of the replated cells from the Sox2-positive population (see Figure 3D). Could the authors please clarify whether the double negative cells represent neural progenitors, and/or alternative cell types? Do the replated cells transiently adopt Tbxt? This would be possible by staining with neural (SOX1), or (pre)somitic mesoderm genes (MSGN1,MEOX1) or adjusting the text to reflect the uncertainty.*

*Reviewer 3*

*The differentiation trajectory described culminates in a double-negative (Sox2-mCherry-negative, Tbxt-GFP-negative) population (lines 246-247). To provide a more complete picture of this fate progression, could the authors perform qPCR for relevant lineage markers to validate the molecular identity of this terminal population?*

We agree with the reviewers that the identity of the generated derivates requires further validation. Since some of the markers proposed by reviewers are not specific to the lineages of interest at early stages (e.g. Nkx1-2 for pre-neural states) and because we are able to generate and cell sort the populations of interest, we propose to do a comprehensive gene expression characterisation by bulk RNAseq of the different NMP-derived differentiated derivatives following the timecourse in Fig.3D. We believe this will provide a comprehensive answer to the points raised by R1, R2 and R3, strengthening our conclusions and giving additional insights into NMP derivatives produced in vitro.

See the proposed experiment layout:

***Neural and lateral plate mesoderm potency of TBXT^GFP^ and SOX2^mCh^ subpopulations***

*Reviewer 2 Optional:*

1. *Altering extrinsic cues (such as RA/CHIR) could clarify how reversible the high TBXT state is as cells progress towards mesoderm. Can you redirect the TBXT-high cells to a neural fate or are they already irreversibly committed?*
2. *Line 283: Flk1 is also expressed in TBXT-low cells. It would be interesting to test whether the TBXT-low cells and the SOX2neg/TBXTpos can generate lateral plate mesoderm or whether this competence to generate lateral plate mesoderm is limited to TBXT-high cells.*

*Reviewer 3:*

*It seems that SOX2 expression also appears heterogeneous from both in vitro differentiation and gastruloid models. Quantifications, and a discussion of this heterogeneity and whether it influences the fate-decision process would be helpful.*

The functional experiments proposed here are interesting and would add value to our exploration of the potency of derivatives coming from NMP populations, as well as the potency of NMPs themselves. We show in Fig3D that Tbxt-GFP positive (sox2-mCherry negative) cells do not generate Sox2-mCherry positive derivatives. Hence, it is unlikely that TBXT^GFP^ single positive cells generate neural derivatives (this will also be unveiled with our bulk RNAseq experiments proposed above). However, we have not challenged our populations to ‘neural only’ extrinsic cues such as RA/SAG (which are also used in Gouti et al. 2014 [10.1371/journal.pbio.1001937](https://doi.org/10.1371/journal.pbio.1001937) to induce neural identities), instead we used FGF/CHIR which is permissive for both mesoderm and neural.

In fact, we propose in our model that TBXT levels (not the ratio of SOX2/TBXT) dictate mesoderm bias and specification while a drop in SOX2 levels is required for a terminal mesoderm specification state. As R3 points out Sox2 heterogeneity in our system, it would also be interesting to test mesoderm vs neural potency in the subpopulation of NMP cells that is Tbxt-high, based on their Sox2 levels (high/low).

To address these points, we propose to do the following cell sorting and differentiation assays, followed by immunostaining for differentiated lineages (neural, mesoderm and lateral plate mesoderm):

Together with the bulk RNA-seq analysis of NMP derivatives, this will convert some of our speculations into data, and both strengthen and provide additional depth to our conclusions.

***Gastruloids and Live imaging***

*Reviewer 3:*

1. *The claims regarding the dynamics of TBXT and SOX2 expression in gastruloids following WNT/NOTCH inhibition (Figure 4B, 4D, 4E) would be more compelling if the authors include supplementary videos of the time-lapse imaging.*

We have in-hand time-lapse videos of DMSO, IWP2 and LY411575 treated gastruloids [[LINK](https://www.dropbox.com/scl/fo/8kevswtujqjvipqwgqyh1/AJdeqkXs1-_RmQc-pp-AOgw?rlkey=9b96kna5ouks4b9phgqgl0q4w&st=zddw6yb3&dl=0)] and will provide them as supplementary files in the format that is appropiate to the journal.

2. *The observation that Sox2 levels increase ~10-15 hours after WNT/NOTCH inhibition is interesting (Figure 4D, 4E). Could the authors discuss this upregulation?*

This is indeed relevant and could be discussed more fully. We will incorporate into our revised discussion the conclusion that WNT or NOTCH inhibition may result in a delayed overproduction of neural cells and that, on specification to neural identity, SOX2 levels may increase. This is entirely consistent with *in vivo* data (Wymeersch et al. 2016 DOI: 10.7554/eLife.10042; and French et al. 2025 DOI: 10.1371/journal.pbio.3003244).

***Validation in vivo***

Reviewer 1:

*At line 172 "The cells posterior to the node expressed only TBXT." Do these cells have low SOX2 expression that is hard to detect? Are these TBXT-positive cells derived from the primitive streak? Would staining with the primitive streak marker TBX6 enable visualization of these distinct cell types, and/or could the authors please label the figure in more detail.*

We have enhanced brightness +100 on the existing Sox2 immunostaining (see Sox2* panel below). Even then, cells posterior to the node remain Sox2 negative/background level at LSEB-NP stages. We have included additional new data on TBX6 and CDX2, showing that these post-nodal cells are TBX6+CDX2+ but lack SOX2 (see examples of the additional data below). We will include this additional data in a new supplementary figure. We will also label the existing figure better to guide the reader, as suggested. We would like to emphasize that the reasoning for performing this time series of stainings was to determine the SOX2/TBXT profile of cells anterior to the node, which will later form NMPs. What we would like to highlight here, for the first time, is that this anterior region is SOX2 positive, and the cells there subsequently acquire TBXT. We related this gene expression sequence *in vivo* to our *in vitro* data when generating NMPs.

*Reviewer 3*

1. *The study's conclusions would be substantially strengthened by more direct validation in an embryonic context. Could the authors quantify endogenous SOX2 and TBXT protein levels of the NMPs in the mouse embryos with immunofluorescence? This would test whether a similar heterogeneity in TBXT expression exists in vivo and whether it correlates with the cells' spatial position.*

This is an important question that we had begun to explore in previous publications. We know that TBXT heterogeneity exists within NMP populations. For example, this has been quantified in past work in our lab (Wymeersch et al. 2016 DOI: 10.7554/eLife.10042; and French et al. 2025 DOI:10.1371/journal.pbio.3003244). Additionally, we have extensively characterized the neural and mesodermal potency of distinct regions of the progenitor zone in the mouse embryo (Cambray et al. 2002 DOI: 10.1242/dev.129.20.4855, Cambray & Wilson 2007 DOI: 10.1242/dev.02877, Wymeersch et al. 2016 DOI: 10.7554/eLife.10042).

Recently, we described in detail the spatial heterogeneity of TBXT and SOX2 (French et al. 2025 DOI:10.1371/journal.pbio.3003244). Importantly, SOX2 and TBXT levels varied along a gradient in ‘pseudospace’- a new term coined to describe how levels of SOX2, TBXT and TBX6 varied in areas known through our fate map experiments to contribute to neurectoderm, mesoderm or both. We have carried out a further analysis of this data to test whether the clonal in vitro data correlates with the individual-cell levels of SOX2 and TBXT in regions of known fate. Our initial analysis in response to reviewer comments highlights that the gradients of SOX2 and TBXT are not opposing, suggesting that these two factors are not simply mutually inhibitory (Panels A and B below, showing vectors representing expressing levels of SOX2 and TBXT). To explore this hypothesis further, we have turned to mutual information (Dubuis et al. 2013 DOI: [10.1073/pnas.1315642110)](https://doi.org/10.1073/pnas.1315642110), as a means to detect non-linear predictive correlations between the single-cell expression of SOX2 and TBXT, and the known fate of subregions of the caudal lateral epiblast. This preliminary analysis indicates that over most of the epiblast, TBXT gives more information than SOX2, except at the midline where cells commit to a mesoderm fate, and SOX2 levels drop (Panels C and D below, showing regions selected for mutual information analyses and their predictive correlations, respectively). This exactly parallels our analysis in vitro and predicts, counterintuitively, that TBXT levels are more important than SOX2 levels in the neuromesodermal-to-neural decision. This new analysis will increase the broad appeal of the manuscript and we propose to refine it further: e.g, the analysis has been done on 6 embryos at 4-6 somite stage, but we have additional embryos, and can also quantify the information provided by TBX6, while further investigating mutual information at specific points of known fate and potency.

*Reviewer 3*
*A key limitation is the study's primary focus on in vitro models. The findings will require further validation in an in vivo context.*

We agree that *in vivo* validation is key. We believe that the addition of the embryo imaging data that we suggest above will provide this.

**Mathematical modelling**
*Reviewer 3:*

*The role of SOX2 in the quantitative model seems underdeveloped. To justify the authors' claim, could the authors analyze their existing imaging data in more detail to disentangle if the absolute TBXT level is essential rather than the ratio of Sox2 to Tbxt is the driving factor for determining NMP fate?*

In our mathematical modelling we show that there is no added structure in the data revealed by using the SOX2/TBXT ratio compared to TBXT alone (Suppl. Fig. 4), and this contributes to our argument that the relatively low, random SOX2 levels are inconsequential to NMP fate decisions. Our suggested incorporation of embryo image analysis will add weight to this argument. If desired, we can additionally include in the supplementary data a representation of the clones in Fig.5C ordered by SOX2^mCh^ levels (see below), showing that no patterns are revealed by SOX2^mCh^ input levels alone.

## 3. Description of the revisions that have already been incorporated in the transferred manuscript

*Reviewer 1 Minor points:*

1. *It would also be important to confirm that IWP2 blocks WNT signalling (by looking at WNT target genes or staining for active beta-cateinin) and that LY411575 blocks NOTCH signalling.*

IWP2 and LY411575 are widely used and well established reagents that block WNT (DOI:*10.1038/nchembio.137)* and NOTCH signalling (DOI: [10.1074/jbc.M311652200](https://doi.org/10.1074/jbc.M311652200)), respectively, we have now added these references to the text. The effect of these inhibitors have been characterized and used at comparable concentrations in studies relating to axial elongation NMPs in the past (inhibitors) (referenced in line 313). In our study, we used these 2 reagents as proof-of-concept to show that our cell line is suitable to track gene expression changes live in gastruloids. Given that we have an effect on axial elongation upon treatment, we believe the experiments proposed here are not necessary in this case.

2. *I would define in the figure legends what the black line in figure 5E represents*

**We have now amended the Figure legend for clarity and added to the text (Line 494-495):***‘*The black line shows a step-function fit to the data, indicating a neural-to-mesodermal lineage transition at ~1745 Tbxt-GFP [AU]’.
We had generated the same analysis as a function of the Tbxt/Sox2 ratio in Suppl. Fig.4D, hence we have also modified its figure legend and defined the black line.

*Reviewer 2:*

1. *Line 310 - Can you comment on the general efficiency in generating elongating gastruloids compared to WT cells and/or previous literature?*

We realise that our Suppl. Fig. 2F, where we addressed this point by looking at timings of elongation in gastruloids generated with our novel double reporter in previously-reported conditions, wasn’t well highlighted in the text. We have added a reference in Line 310-311 to the previous publication we based our protocol on, and which also used a TBXT-GFP reporter line to monitor elongation. **We now emphasise in the text that our gastruloids proceed with similar timings to those generated in other laboratories** (van den Brink et al., 2014).

2. *Line 236 - Add "at constant SOX2 levels" (when comparing orange and yellow populations).*

We agree with the reviewer that SOX2 levels remain constant across our two NMC subpopulation in our analyses, as seen from our flow cytometry data (reporter) but also our gene expression analyses for endogenous Sox2 (Fig 3B). We had already highlighted in our text that the NMC population ‘displayed a relatively constant Sox2-mcherry fluorescence (line 214). However, **we have now added ‘at constant Sox2 levels’ in line 217,** to clearly specify that the Tbxt-low and Tbxt-high populations we will be comparing are at a constant Sox2 level.

3. *Fig5C and E take time to understand. Potentially expanding the figure legend slightly could be helpful to the reader.*

We have now **amended the figure legend** to explain more clearly:

**5C.** Summary of the total lineage output of colonies derived from single cell deposition of sorted Sox2^mCh^pos;Tbxt^GFP^high or Sox2^mCh^pos;Tbxt^GFP^low cells. Colonies were immunostained for endogenous SOX2 and TBXT proteins, and analysed at 8 days post-sorting. Stripes denote the presence of at least one double positive (DP) cell, and colours denote their derivatives: Sox2 pos (magenta), TBXTpos (green), a mixture of the two single positive cell types (blue), or only double negative cells (grey) were detected, with n being the number of colonies scored for each substrate.

**5E.** Individual colony lineage output as a function of the initial Tbxt^GFP^ levels in the founder NMP. Each dot represents the lineage output from one colony, coloured by the substrate used: matrigel (dark green), geltrex (light green), gelatin (pink) and fibronectin (fuchsia). The data was segregated in bins of equal number of colonies and the Tbxt^GFP^ level on the horizontal axis is the average of input levels of Tbxt-GFP levels per bin. The black line shows a step-function fit to the data, indicating a neural-to-mesodermal lineage transition at ~1745 Tbxt-GFP [AU].

4. *Line 864 - refer to Fig3A*

We thank the reviewer for spotting this mistake. **We have now corrected the text and added Fig3A.**

5. *Line 350 - The link with testing the different substrates is a bit abrupt. Please can you make it clearer by modifying the text to explain the hypothesis being tested here.*

**We have now introduced why we tested different matrices** in line 351 by adding : ‘We hypothesised that on top of Tbxt-expression levels, extracellular matrices mightalso impact cell fate decisions’

6. *Line 363 - Could you comment in relation to what happens in the embryo to future mesoderm progenitors that seem to have a more motile phenotype compared to neural progenitors (eg Romanos et al 2021 PMID: 34607629)?*

This is an interesting point. The observations of Romanos et al., 2021 on the lineage outcome of SOX2 and TBXT positive cells suggest increased motility of TBXT-high cells. In our clonal studies, we see increased spreading in Matrigel, fibronectin and Geltrex. we are plating individual cells into different matrices but we are not monitoring their cellular behaviour in terms of motility or colony formation dynamics. We have now added text in line 369-373 reflecting this.

7. *it is not entirely clear in this version of the manuscript how these advances relate with previous studies that have highlighted critical roles mediated by Sox2 and its level of expression (including the work of Koch et al 2017 - PMID: 28826820 and Blassberg et al PMID: 35550614). We expect that a broader discussion will in turn broaden the general interest and value of the work to a wider readership.*

We have added text to the existing discussion to slightly expand on our reinterpretation of the literature in the light of our results (line 561-562). However, we believe that the mutual information analysis of embryo imaging data will provide us with further insights and will substantially rework and expand this section of the discussion as suggested once this is complete. In the reworked discussion we will deal more fully with the proposition that TBXT and SOX2 are mutually inhibitory, and discuss why the ChIP-seq data that suggested mutual inhibition (Koch et al. 2017 DOI: 10.1016/j.devcel.2017.07.021) is open to several interpretations. In short, it is claimed that TBXT represses neural targets based on the comparison of ChIP-seq data with bulk RNA-seq of wildtype and TBXT mutant embryos. In these mutant embryos, mesoderm is underproduced and neurectoderm is unaffected, meaning that neural tissue is overrepresented, not necessarily that neurectoderm genes are normally repressed by TBXT. The converse analysis in SOX2 mutant embryos was not performed.

*Reviewer 3*

3. *Scale bars for micrographs are missing in Figure 1B, Suppl. Fig. 1C, and D.*

We have now added scale bars and made a minor staging correction to the legend of Figure 1.

4. *In lines 322-324, the authors conclude that Tbxt-cells are the driving cells. Please elaborate on the interpretation that this is a cell-autonomous effect driven by TBXT levels.*

In this concluding sentence, we stated ‘the rapid Tbxt^GFP^ signal downregulation coupled with slowdown of axis elongation in response to WNT or NOTCH inhibition suggests AP axis elongation is driven predominantly by *Tbxt-*expressing cells.’ The experiments are not imaged at single cell resolution so are agnostic about cell autonomy of the effect, and therefore we did not comment on this aspect in the results. However, our clonal analysis clearly shows that TBXT is acting cell-autonomously in decision-making. In the discussion, to make sure we do not imply a cell-autonomous effect in the TBXT response to WNT/NOTCH, we have changed the last paragraph (line 581), from ‘Our study indicates that this works at the single cell level..’. to read ‘Our study suggests that cells sense cues in their environment (e.g. WNT, NOTCH), respond by tuning TBXT up or down, which in turn influences the differentiation outcome at the single cell level.’

## 4. Description of analyses that authors prefer not to carry out

Reviewer 3

2. *To support the authors' claim that the reporter line recapitulates endogenous protein expression in vivo (lines 121-128), please include a control immunostaining of wild-type embryo for SOX2 and TBXT to compare expression patterns side-by-side with those embryos shown in Figure 1B, Suppl. Fig. 1C, D. To substantiate the claims regarding cellular expression patterns within the embryo (line 125-128), the use of higher-resolution imaging, such as confocal microscopy, is recommended.*

We believe the expression patterns of these 2 genes are well-described in the literature. For example, see Avilion et al., 2003 DOI: 10.1101/gad.224503; and Kispert et al., 1995 DOI: 10.1006/dbio.1995.1090 for Sox2 and Tbxt, respectively. See also Wymeersch et al. 2016 DOI: 10.7554/eLife.10042; and French et al. 2025 DOI:10.1371/journal.pbio.3003244 for expression patterns of Sox2 and Tbxt in the posterior/tail bud region.

*Reviewer 2 Optional*
*Line 324: The lack of elongation in gastruloids following the inhibition of WNT/NOTCH is clear. Do the authors expect that the reintroduction of TBXT would rescue axis elongation in the WNT/NOTCH inhibited gastruloids?*

We believe that doing this experiment is beyond the scope of our current paper and would perhaps detract from the linear narrative. We have reworded the final sentence in our discussion to suggesting this as a further investigation made possible by the STR-KI cell line.
