# Supplementary material for: Brachyury expression levels predict lineage potential and axis-forming ability of in vitro-derived neuromesodermal progenitors
Source: PLoS Biol. 2026 Aug 25;24(8):e3003960. doi: 10.1371/journal.pbio.3003960 (PMC13533433; doi:10.1371/journal.pbio.3003960)
Supplement: Supplementary file 4 [file pbio.3003960.s018.docx]

Comments from the academic editor:

1.  A key conclusion is that Sox2 levels are not well correlated with fate choice, while Tbxt levels are. A crucial issue that needs to be addressed is how this finding relates to prior studies of the mechanism governing NMP fate choice, in particular Koch et al 2017 and Gouti et al 2017. Both of these studies have published putative gene regulatory networks underlying the fate decision. The current manuscript should spell out more explicitly what alternative mechanism/ interaction network their findings suggest. The authors seem to hint at the possibility that Tbxt does not directly repress Sox2, which suggests that instead Sox2 is only (or much more strongly) repressed by Tbx6, representing a refinement of the existing models by Koch and Gouti. A direct test of this hypothesis will greatly strengthen the manuscript. For instance, if Sox2 is not expected to contribute to the fate bias, then the Tbxt-driven bias should be preserved in conditions of RA/SAG. The authors could test this by expanding on the experiment they propose in the revision plan to also include Tbxt-Low populations in the section ‘Neural and lateral plate mesoderm potency of TBXTGFP and SOX2mCh subpopulations’. The figure presented in the revision plan under “mathematical modeling” should also be included in the revised manuscript.

*We attempted the excellent suggestion to investigate the Tbxt-driven bias in RA/SAG conditions (as in Gouti et al 2014) after sorting populations of differing Tbxt/Sox2 reporter expression levels. However, multiple repeats of this experiment were unsuccessful due to, presumably, a high toxicity of RA/SAG in our culture conditions after NMP-purification. Our cultures resulted in high cell death when plating sorted populations and treating them with RA/SAG, which we could not resolve. We have instead extensively analysed the embryo imaging datasets available to us, which provides an in vivo, spatially-anchored second test that supports the dominant role of TBXT compared to SOX2 in predicting cell fate. This data is now included in a new section and new figures Fig 6 and Suppl. Fig 5-6. We have also expanded the discussion to suggest how our results might be reconcilable with those of previous authors (including ourselves!) and where we propose novel findings. Of note, we have included Gouti et al. 2017, in our introduction to this section of the results, but because it deals with a GRN model that assumes and does not directly test TBXT versus SOX2 function, we felt it was not relevant in the discussion.*

*We have now included our in vitro clonal colonies ordered by Sox2-mcherry in Suppl Fig3 as suggested.*

2.  The authors should provide a clearer representation of the two thresholds and a clearer distinction of the biased vs committed state as a function of Tbxt level. Fig 5E shows the data related to the first threshold (for bias); the second, mesoderm commitment threshold is not clearly shown – in fact, my understanding is that committed cells are identified as Sox2 negative, rather than having even higher Tbxt levels. The relevance of Tbxt and Sox2 levels should be clarified for both of these transitions.

*The commitment threshold that happens at higher levels of TBXT is not precisely defined by our experiments: we retrospectively tracked sorted populations to their clonal history, without indexing. Therefore, we are less certain about the relative SOX2 and TBXT levels that define the commitment threshold. We think it’s unlikely to be zero SOX2, and we quantified the percentage of cells in this gate that falls into the 50% percentile of Sox2-mcherry in each of our experiments (Suppl Fig 3B). We believe the current diagram in figure 5 does not overclaim or misrepresent the findings, but are open to further comment.*

3.  The manner in which the mathematical model is presented and interpreted is currently misleading and needs to be revised. The ‘model’ presented in the manuscript is not mechanistic and does not have predictive value with respect to the role of Tbxt levels on fate choice. What it does is simply infer the differentiation rates from data, in other words this is an alternative way to represent the observed fate choices in the different conditions. Statements such as the one on line 410, that the model is used to determine dependence on levels, or line 444 ‘to gain mechanistic insight’, or line 470 ‘clonal analysis and mathematical modeling’ – are therefore misleading. I would suggest calling it ‘estimation’ or ‘inference’ of differentiation rates from data.

*Thanks for this. We accept the criticism and have modified the text accordingly, substituting modelling with estimation/ inference throughout, including the text mentioned above. We have left some instances of the verb ‘to model’, especially in the methods section, that we believe are correct (meaning ‘to extract a mathematical representation of the data’) but believe we have now removed any overclaim in the results and discussion.*

Reviewer #1 (Evidence, reproducibility and clarity (Required)):

Minor points:

1. The reporter system is based on stable fluorescent proteins, whose half-life is generally much longer than the endogenous proteins. This could generate a discrepancy between expression of reporters and the endogenous proteins.

I find this relevant for Tbxt, as it is very clear that Tbxt reporter levels dictates the differentiation propensity, but I wonder whether, Tbxt low cells actually express TBXT protein or not. It might be the case that only a fraction of Tbxt low cells actually express TBXT protein, or none. 
It would enough to sort the populations showed in Figure 3A and perform immunostaining for endogenous SOX2 and TBXT. This could reveal even better correlations between their levels and cell behaviour.

*We thank the reviewer for this point. Our original validation data demonstrated a strong correlation between the fluorescent reporters and their respective endogenous proteins. For example, in embryos, GFP is correctly localised to the nascent mesoderm (Fig 1B and SFig 1C), while mature mesoderm anterior to the primitive streak shows no GFP expression, consistent with the known spatio-tempral dynamics of TBXT. Most strikingly, Fig 1C shows that endogenous SOX2 and TBXT protein levels correlate closely with their respective reporters, and we have now included the R-squared values of these quantifications (line 140-41). Moreover, in chimeras made with STR-KI cells, areas where expression of endogenous protein decreases (specifically, in the nascent mesoderm for SOX2 and in the presomitic mesoderm for TBXT, fluorescence levels are low – see Fig S1C).*

*Regarding the concern about fluorescent protein stability, our experiments in gastruloids demonstrate that, despite the expected stability of H2B-GFP, Tbxt-GFP fluorescence decreases within 3 hours of inhibitory treatment (Fig 4E). This suggests that GFP fluorescence does not persist once inductive signals are reduced, indicating that reporter levels most likely reflect active expression rather than protein accumulation.*

*To further address the reviewers concern and quantify reporter-endogenous protein correlation at the single cell level, we have now supplemented our immunostaining of fluorescent cells with intracellular flow cytometry to rigorously detect expression levels protein (Fig 1C, SFig1B). The result agrees with our image analysis and supports highly-correlated fluorescent reporter and endogenous protein levels.*

2. In the experiments in which IWP2 and LY411575 are used, I would suggest to asses cell viability, as the two inhibitors could induce toxicity. Staining for cleaved caspase-3 or a TUNEL assay would be enough.
It would also be important to confirm that IWP2 blocks WNT signalling (by looking at WNT target genes or staining for active beta-cateinin) and that LY411575 blocks NOTCH signalling.

*IWP2 and LY411575 are widely used and well-established specific inhibitors of WNT (DOI: 10.1038/nchembio.137) and NOTCH signalling (DOI: 10.1074/jbc.M311652200) respectively, and have been extensively characterized at comparable concentrations in studies of axial elongation and NMPs in the past (referenced in line 386-88). In our study, we used these 2 reagents as proof-of-concept to show that our cell line is suitable to track gene expression changes live in gastruloids. Given that we have an effect on axial elongation upon treatment (impaired, not absent, elongation), which is consistent with the documented roles of  WNT and NOTCH signalling in the process, but not an absence or collapse of gastruloids themselves, or complete elimination of Sox2+ cells, which would suggest toxicity and cell death, we consider the experiments proposed here are not necessary in this case.*

3. I would define in the figure legends what the black line in figure 5E represents.

*We have now amended the figure legend for clarity and added to the text (Line 575-76): ‘The black line shows a step-function fit to the data, indicating a neural-to-mesodermal lineage transition at ~1745 Tbxt-GFP [AU] (bias switch at the vertical)’. We had generated the same analysis as a function of the Tbxt/Sox2 ratio in Suppl. Fig 4D, hence we have also modified its figure legend and defined the black line.*

**Referees cross-commenting**

I agree with Reviewer #2 comment about "Further validation of the generated derivatives" by staining for additional markers.

*This is addressed below.*

I also made a comment related to GFP stability, as Reviewer #2 did (i.e. The low versus high TBXT cells may reflect early versus late TBXT expressing cells ).

*We believe we addressed this above.*

Reviewer #2 (Evidence, reproducibility and clarity (Required)):

MAJOR COMMENTS:

With respect to the mESC differentiation into NMPs (using an established protocol from Gouti et al 2014), after Day 3, cells are cultured in conditions where they are exposed to both CHIR and FGF for a further two days. As these extended CHIR/FGF conditions don't appear to be characterised by Gouti et al., 2014, what proportions and progenitors are generated in the dish under these conditions? The loss of SOX2 by day 5 suggests mesodermal progenitors are the main derivatives but further characterization (eg Meox1/Msgn1) would be needed to verify this claim. Further validation of the generated derivatives would also be useful in the re-plating experiments (Figure 3) to test whether the double negative cells are transitioning to a mesodermal (eg Meox1/Msgn1) or neural derivative (eg Sox1/Pax6). Similarly, at day 5 and 6 of the differentiation, there appears to be a loss of Sox2 expression in some of the replated cells from the Sox2-positive population (see Figure 3D). Could the authors please clarify whether the double negative cells represent neural progenitors, and/or alternative cell types? Do the replated cells transiently adopt Tbxt? This would be possible by staining with neural (SOX1), or (pre)somitic mesoderm genes (MSGN1,MEOX1) or adjusting the text to reflect the uncertainty.

*We agree that further characterisation of the generated derivatives was needed and have addressed these important points by carrying out bulk RNA-sequencing of sorted populations along the extended culture period (+7d post NMP induction), starting from a pure Sox2/Tbxt co-expressing population to double negative cell derivatives (Fig 3D-G).*

*We find that Tbxt-derived populations after extended culture express predominantly mesoderm markers (e.g. Meox1, Tbx6 , Tcf15 early and Osr1, Meox2 later), while Sox2-derived populations have a more mixed phenotype in FGF and Chiron. Plating these Sox2-derived populations (which are Pax6 and Sox2 positive) in FGF alone, shows neural lineage differentiation (expressing genes such as Gsx1, Msx3, Olig3, and Phox2b). Interestingly, these observations are consistent with the idea that WNT pathway activation in early NMP derivatives is incompatible with progression to a differentiated neural phenotype (but does not block early neural progenitor differentiation). Together, our new data confirm that the double-negative population does not represent an alternative uncharacterised cell type and shows that our new reporter cell line is useful for capturing intermediate progenitors transitioning towards either mesoderm or neural fates that can vary depending on the signalling context.*

At line 172 "The cells posterior to the node expressed only TBXT." Do these cells have low SOX2 expression that is hard to detect? Are these TBXT-positive cells derived from the primitive streak? Would staining with the primitive streak marker TBX6 enable visualization of these distinct cell types, and/or could the authors please label the figure in more detail.

*Thanks to this reviewer’s comment we have now improved this section. We have added an additional panel with enhanced brightness +100 on the existing Sox2 immunostaining (see Sox2* in new Fig 2B). Even then, cells posterior to the node remain Sox2 negative/background level at LSEB-NP stages. We have included additional new data on TBX6, to highlight the primitive streak region as suggested by the reviewer. Our new panel shows that these post-nodal cells are TBX6+ but lack SOX2. We have also included a video 3D-reconstruction of the Node Streak Border region in embryos before and after the LB/EHF stage, highlighting when SOX2 expressing cells gain TBXT expression (Suppl video 1). We also labelled the existing figure better to guide the reader (namely added NSB labels), as suggested.*

*We would like to emphasize that the reasoning for performing this time series of stainings was to determine the SOX2/TBXT profile of cells anterior to the node, which will later form NMPs (as seen from our own lineage trace and Forlani et al 2003, compiled in Fig 2D). What we would like to show here, for the first time, is that this anterior region is SOX2 positive, and cells there subsequently acquire TBXT. We related this gene expression sequence in* *vivo to our in vitro data when generating NMPs.*

Could the authors please comment on the design of the reporter system in a bit more detail? For example, please clarify the necessity to generate a TBXT reporter that includes a H2B-GFP, unlike Sox2, which does not include a H2B. Can the authors distinguish between an increase in the threshold of TBXT, versus an increase detected due to the stability of the H2B-GFP? The low versus high TBXT cells may reflect early versus late TBXT expressing cells. Are the changes in TBXT expression (eg Supplementary Figure 2) significantly changed between the low versus high GFP populations? Additionally, why is the level of GFP similar across low and high GFP populations in Sup Fig 2? Could the in vivo data be used to quantify differences in TBXT (similar to what has been shown previously in Ivanovitch et al., 2021 PMID: 33999917)?

*We have shown here that using a single H2B-tagged reporter combined with a whole-cell mCherry reporter allows reliable single-cell discrimination of both signals while minimising potential disruption to normal cellular function. We have clarified this in the text (line 820-21).*

*The concern regarding H2B-GFP stability and whether low versus high Tbxt-GFP populations reflect true expression differences versus protein accumulation is addressed in detailed in our response to Reviewer 1 above. Briefly, our intracellular flow cytometry data (Fig. 1C, SFig. 1B) provide excellent validation of our reporter system by directly measuring endogenous protein levels rather than relying on mRNA levels alone. Supplementary Figure 2 (day 4 data) and Figure 3 (day 3 data) shows broadly consistent trends between reporter and endogenous mRNA levels across populations, though we recognise that bulk measurements have inherent limitations; we therefore consider the intracellular flow cytometry data to be the most rigorous validation of reporter fidelity in our system. Furthermore, our gastruloid experiments show that GFP fluorescence decreases within 3 hours of inhibitory treatment (Fig 4E), arguing against persistent accumulation of stable protein driving the low/high distinction.*

*In vivo quantification of endogenous TBXT levels across different NMP regions is addressed in our new section 6 in the MS ‘TBXT and SOX2 in vivo spatial patterns provide separate information to NM fated regions’ and accompanying Fig 6, Suppl fig 5,6.*

Altering extrinsic cues (such as RA/CHIR) could clarify how reversible the high TBXT state is as cells progress towards mesoderm. Can you redirect the TBXT-high cells to a neural fate or are they already irreversibly committed?

*As noted above in comments to the editor, we tried in multiple experiments to culture sorted subpopulations in RA/SAG in different combinations/concentrations, and found that treatment with RA or RA/SAG (as in Gouti et al) yielded high cell death, suggesting that it is toxic to these purified cells in our culture conditions. Nevertheless, our existing data provide indirect evidence that TBXT-high cells are already committed to a mesodermal fate. First, our qPCR data at day 3 (Fig 3B) and day 4 (Supp Fig 2A-B) show that Tbxt-high/Sox2-positive NMP populations express high levels of* Tbx6*, a marker of mesodermal commitment. Second, in our replating experiments (Fig 3D), Tbxt-GFP positive/Sox2-mCherry negative cells fail to generate Sox2-mCherry positive derivatives even under permissive FGF/CHIR conditions, and their gene expression profile is consistent with a committed mesodermal population. Together, these data suggest that Tbxt-high cells are already committed to a mesodermal fate. How and at what point cells commit to their fates in vitro will be an interesting area for further study, but we believe the lack of these experiments do not alter the conclusions of the present study.*

Line 283: Flk1 is also expressed in TBXT-low cells. It would be interesting to test whether the TBXT-low cells and the SOX2neg/TBXTpos can generate lateral plate mesoderm or whether this competence to generate lateral plate mesoderm is limited to TBXT-high cells.

*We thank the reviewer for this suggestion. We cultured a more refined gating of the d3 NMP culture subpopulations in BMP4 and performed Flk1 immunostaining. We find indeed that the d3 TBXT-low populations (as well as the TBXT-negative population) produce significantly more Flk1 than the TBXT-high ones, where Flk1 expression is negligible. This suggests that there is a window for LPM endothelial induction in NMPs, shortly after cells gain TBXT expression, and, consistent with our new RNAseq data (Fig. 3D-G), the mesoderm formed by the bulk of the NMC population is predominantly paraxial (for example, it expresses Tbx6, Dll1, Dll3, Tcf15, Meox1 and Meox2). New Suppl. Fig 3 D-E shows the exact gating within the Sox2/Tbxt reporter co-expressing population.*

Line 324: The lack of elongation in gastruloids following the inhibition of WNT/NOTCH is clear. Do the authors expect that the reintroduction of TBXT would rescue axis elongation in the WNT/NOTCH inhibited gastruloids?

*This would be a reasonable prediction. We have not carried out these experiments, but our previous in vivo data on TBXT overexpression (Wilson et al. Development 1995) are relevant: the levels of TBXT are quite critical for a cell’s decision to remain NMP/move away as mesoderm, so one would imagine that either overproduction or underproduction of TBXT would shorten axis elongation.*


MINOR COMMENTS:

Figure 3B - The SOX2+/TBXT- population only shows a moderate level of SOX1 by RT-qPCR. Using pre-neural markers (e.g. NKX1-2) might be helpful here to show progression towards a neural progenitor identity.

*Nkx1-2 itself is expressed in NMPs as well as emergent neurectoderm, but our new RNAseq data shows expression of additional neural markers in Sox2^mch^ positive cells. The low expression of Sox1 was most likely symptomatic of our eventual conclusion from the RNAseq data analysis: culture in FGF-CHIR allows entry of double-positive cells to the neural lineage, but neural differentiation marker expression requires the removal of CHIR (Fig. 3F-G).*

Line 310 - Can you comment on the general efficiency in generating elongating gastruloids compared to WT cells and/or previous literature?

*We realise that our Suppl. Fig. 2F, where we addressed this point by looking at timings of elongation in gastruloids generated with our novel double reporter in previously reported conditions, wasn’t well highlighted in the text. We have added a reference in line 381-83 to the previous publication we based our protocol on, and which also used a TBXT-GFP reporter line to monitor elongation. We now emphasise in the text that ‘our gastruloids proceed with similar timings to those generated in other studies (van den Brink et al., 2014)’.*

Line 236 - Add "at constant SOX2 levels" (when comparing orange and yellow populations)

*We added in line 239-40: ‘To test this, we divided the NMC-like population into low-TBXT (Sox2^mCh^pos/Tbxt^GFP^low) and high-TBXT (Sox2^mCh^pos/Tbxt^GFP^high) expressing subpopulations, where SOX2 levels were relatively constant.*

Figure 5

Fig5C and E take time to understand. Potentially expanding the figure legend slightly could be helpful to the reader.

*We agree that the figure is information dense. We have expanded the legend as suggested (line 552-81) to better indicate important features.*

Supplementary Figure 2 Line 864 - refer to Fig3A

*Thank you for spotting this. We have amended this reference in line 1176 in the legend of Suppl. Fig 2.*

Line 350 - The link with testing the different substrates is a bit abrupt. Please can you make it clearer by modifying the text to explain the hypothesis being tested here.

*We have now introduced why we tested different matrices in line 426 by adding : ‘We hypothesised that on top of Tbxt-expression levels, extracellular matrices might also impact cell fate decisions’*

Line 363 - Could you comment in relation to what happens in the embryo to future mesoderm progenitors that seem to have a more motile phenotype compared to neural progenitors (eg Romanos et al 2021 PMID: 34607629)?

*This is an interesting point, we thank the reviewer for highlighting it. The observations of Romanos et al., 2021 on the lineage outcome of SOX2 and TBXT positive cells suggest increased motility of TBXT-high cells. In our clonal studies, we see increased spreading in Matrigel, fibronectin and Geltrex. Here we are plating individual cells into different matrices but we are not monitoring their cellular behaviour in terms of motility or colony formation dynamics. We have now added text in line 445-49 reflecting this. In the future, we plan to look at cell motility of TBXT, SOX2 and double positive populations with our reporters in monolayer.*

Reviewer #2 (Significance (Required)):

In this work, the authors have explored how the dynamics of SOX2/TBXT impact the decision process of neuromesodermal progenitors (NMPs). They have engineered and validated a novel dual fluorescent reporter ESC line to track SOX2 and TBXT. The work combines in vitro, in vivo and modelling approaches to understand how NMPs make decisions - a highly relevant and important question for multiple fields spanning developmental and quantitative biology, and the engineering of cell types in vitro from pluripotent cells. The authors propose a critically important finding: that discrete thresholds of TBXT influence the outcome of NMPs. However, further clarification is required to solidify these claims (discussed above).

*We hope the clarifications provided (responses above) are satisfactory.*

The data generated from this study also suggests that in contrast to Tbxt, the level of Sox2 does not appear to impact the NMP fate decision. While these are interesting and important findings, it is not entirely clear in this version of the manuscript how these advances relate with previous studies that have highlighted critical roles mediated by Sox2 and its level of expression (including the work of Koch et al 2017 - PMID: 28826820 and Blassberg et al PMID: 35550614). We expect that a broader discussion will in turn broaden the general interest and value of the work to a wider readership.

*We have expanded the discussion on this topic (lines 760-85) to contextualise our in vitro clonal and embryo image analysis data that we think support indpendnt roles for TBXT and SOX2 in fate decisions.*


Reviewer #3 (Evidence, reproducibility and clarity (Required)):

MAJOR COMMENTS

The study's conclusions would be substantially strengthened by more direct validation in an embryonic context. Could the authors quantify endogenous SOX2 and TBXT protein levels of the NMPs in the mouse embryos with immunofluorescence? This would test whether a similar heterogeneity in TBXT expression exists in vivo and whether it correlates with the cells' spatial position.

*This is an important question that we had begun to explore in previous publications. We know that TBXT heterogeneity exists within NMP populations. For example, this has been quantified in past work in our lab (Wymeersch et al. 2016 DOI: 10.7554/eLife.10042; and French et al. 2025 DOI:10.1371/journal.pbio.3003244). Additionally, we have extensively characterized the neural and mesodermal potency of distinct regions of the progenitor zone in the mouse embryo (Cambray et al. 2002 DOI: 10.1242/dev.129.20.4855, Cambray & Wilson 2007 DOI: 10.1242/dev.02877, Wymeersch et al. 2016 DOI: 10.7554/eLife.10042). Recently, we described in detail the spatial heterogeneity of TBXT and SOX2 (French et al. 2025 DOI:10.1371/journal.pbio.3003244).*

*Thanks to the reviewer’s comment, we have carried out a further extensive analysis of this data to test whether the clonal in vitro data correlates with the individual-cell levels of SOX2 and TBXT in regions of known fate in vivo. The results are now included in a new section in the MS ‘6. TBXT and SOX2 in vivo spatial patterns provide separate information to NM fated regions’ and accompanying Fig 6, Suppl fig 5,6.*

*In brief, our analyses highlight that the gradients of SOX2 and TBXT are not opposing in the epiblast, suggesting that these two factors are not simply mutually inhibitory. To explore this hypothesis further, we have turned to mutual information and gradient precision analysis (Dubuis et al. 2013 DOI: 10.1073/pnas.1315642110 and Haroush et al., 2023 DOI: 10.1016/j.devcel.2023.10.001), as a means to detect non-linear predictive correlations between the single-cell expression of SOX2 and TBXT, and the known fate of subregions of the caudal lateral epiblast. This analysis indicates that over most of the epiblast, TBXT provides much of the predictive power for cell fate decisions and displays highly precise gradients (Fig 6G). In contrast, SOX2 forms a shallow, predominantly anterior-posterior, gradient with no power to independently predict NM to M decisions, except at the node-streak-border. Furthermore, only at the midline do the SOX2/TBXT gradients approximate the ‘antagonism’ model, and even here, they reach maximal precision at different locations. Thus, we conclude that, as in the in vitro clonal analysis (Fig 5), SOX2 and TBXT do not predominantly fit a mutually-repressive model in vivo either, and TBXT levels are generally more predictive of NMP fate decisions than SOX2 ones.*

The role of SOX2 in the quantitative model seems underdeveloped. To justify the authors' claim, could the authors analyze their existing imaging data in more detail to disentangle if the absolute TBXT level is essential rather than the ratio of Sox2 to Tbxt is the driving factor for determining NMP fate? 

*In the same analysis as the point raised above, we have done this in embryos, where we know the fate of caudal epiblast regions. This careful image analysis supports the conclusion from the monolayer clonal analysis over most of the epiblast. The exception to this statement is that at the posterior node-streak border and the primitive streak midline, there is more evidence for a role of SOX2 and a possible antagonistic relationship, suggesting an overall dominant role of TBXT with local deviations from this general rule.*

It seems that SOX2 expression also appears heterogeneous from both in vitro differentiation and gastruloid models. Quantifications, and a discussion of this heterogeneity and whether it influences the fate-decision process would be helpful.

*We have now included a plot of the clonal data ordered by SOX2 input expression (Suppl. Fig 3C). As in the mathematical inference, we see no fate pattern dictated by heterogeneity in SOX2. We added a sentence to the text (line 482-483) to emphasize this point.*

To support the authors' claim that the reporter line recapitulates endogenous protein expression in vivo (lines 121-128), please include a control immunostaining of wild-type embryo for SOX2 and TBXT to compare expression patterns side-by-side with those embryos shown in Figure 1B, Suppl. Fig. 1C, D. To substantiate the claims regarding cellular expression patterns within the embryo (line 125-128), the use of higher-resolution imaging, such as confocal microscopy, is recommended.

*The expression of SOX2 and TBXT has been extensively used to document NMCs in wild-type mouse embryos. For example, see Avilion et al., 2003 DOI: 10.1101/gad.224503; and Kispert et al., 1995 DOI:*

*10.1006/dbio.1995.1090 for Sox2 and Tbxt, respectively. See also Wymeersch et al. 2016 DOI:*

*10.7554/eLife.10042; and French et al. 2025 DOI:10.1371/journal.pbio.3003244 for expression patterns of Sox2 and Tbxt in the posterior/tail bud region. In our manuscript here we show confocal immunofluorescence images of SOX2/TBXT in Fig.2, in wild-type embryos at a slightly earlier stage (6 somite versus 2 somite) than those in Fig 1 and Suppl Fig1. Comparably staged confocal images are found in Wymeersch et al. eLife 2016. Unfortunately, our confocal images of the chimeras are not of sufficient quality to include in the manuscript.*

The differentiation trajectory described culminates in a double-negative (Sox2-mCherry-negative, Tbxt-GFP-negative) population (lines 246-247). To provide a more complete picture of this fate progression, could the authors perform qPCR for relevant lineage markers to validate the molecular identity of this terminal population?

*We thank the reviewer for this suggestion. We have answered this question by extending the analysis in Fig.3C where we followed sorted populations over time. We sorted NMPs, replated and then sorted Sox2^mCh^ and Tbxt^GFP^ single positive populations. These were then cultured for an extended period of 7 days, sorted once more and individual subpopulations subjected to bulk RNA-seq (Fig.3D). This shows that the Tbxt^GFP^ positive population (which universally makes double negative cells after 7 days), expresses mesoderm markers. In our first iteration of this experiment (not shown), all populations were cultured only in FGF/CHIR to minimise variables. However, the Sox2^mCh^ population did not show any enrichment for neural differentiation-specific markers in these conditions. Only when we plated Sox2^mCh^ single positive cells in media without CHIR did we see the expected upregulation of neural markers, and only in the population forming the majority of Sox2^mCh^-derived cells, which remained Sox2^mCh^ positive. The few Sox2^mCh^-derived double negative cells (in FGF/CHIR or FGF alone) did not upregulate neural-specific markers. This suggests that the predominant fate of Sox^mCh^ cells in these conditions is to self-renew as early neural progenitors, whose neural differentiation is likely blocked by CHIR. Thus, the double negative population formed without sorting is most likely constituted by a large TBXT-derived mesodermal majority and a minority Sox2-derived population of uncertain lineage.*

MINOR COMMENTS

Scale bars for micrographs are missing in Figure 1B, Suppl. Fig. 1C, and D.

The claims regarding the dynamics of TBXT and SOX2 expression in gastruloids following WNT/NOTCH inhibition (Figure 4B, 4D, 4E) would be more compelling if the authors include supplementary videos of the time-lapse imaging.

*We apologise for the omission and have included scale bars in the figures. We also include examples of time-lapse imaging in gastruloids following DMSO, IWP2 and LY411575 treatment.*

In lines 322-324, the authors conclude that Tbxt-cells are the driving cells. Please elaborate on the interpretation that this is a cell-autonomous effect driven by TBXT levels.

*In this concluding sentence, we stated ‘the rapid TbxtGFP signal downregulation coupled with slowdown of axis elongation in response to WNT or NOTCH inhibition suggests AP axis elongation is driven predominantly by Tbxt-expressing cells.’*

*Our gastruloid experiments are not imaged at single cell resolution, so are agnostic about cell autonomy of the effect, and therefore we did not comment on this aspect in the results. However, our clonal analysis shows that TBXT is acting cell-autonomously in decision-making in clonally plated cells. In the discussion, to make sure we do not imply a cell-autonomous effect in the TBXT response to WNT/NOTCH, we have changed the last paragraph (line 804), from ‘Our study indicates that this works at the single cell level..’. to read ‘Our study suggests that cells sense cues in their environment (e.g. WNT, NOTCH), respond by tuning TBXT up or down, which in turn influences the differentiation outcome at the single cell level.’*

The observation that Sox2 levels increase ~10-15 hours after WNT/NOTCH inhibition is interesting (Figure 4D, 4E). Could the authors discuss this upregulation?

*This is indeed interesting. We now incorporate into our revised discussion the speculation that WNT or NOTCH inhibition may result in a delayed overproduction of neural cells or that, on specification to neural identity, SOX2 levels may increase (line 396-398). This is entirely consistent with in vivo WNT and NOTCH pathway manipulation data (Wymeersch et al. 2016 DOI: 10.7554/eLife.10042; and French et al. 2025* DOI: 10.1371/journal.pbio.3003244, respectively).*The late upregulation of Sox2 in our gastruloids could further suggest the effects are downstream of the depletion of Tbxt-expression/Tbxt + cells, but not a direct consequence. A concrete conclusion would require further experiments that we consider outside the scope of our work presented here.*

Reviewer #3 (Significance (Required)):

GENERAL ASSESSMENT

In the development of a novel dual Sox2/Tbxt reporter cell line, which provides a powerful tool for quantitatively understanding the dynamics of cell fate specification during gastrulation and potentially in other developmental contexts. However, a key limitation is the study's primary focus on in vitro models. The findings will require further validation in an in vivo context.

*We hope that our quantitative analysis of immunostained* *embryos, which supports the in vitro findings, is helpful to convince the reader of the key role of TBXT*.

ADVANCE 

This study provides a technical advance that provides a new resource available to the field for stem cell and developmental biology.

*We are grateful for this assessment of the usefulness of the tool and also hope that the new data moves the paper in the reviewer’s mind from simply technical to one of biological importance.*
