# Supplementary material for: Brachyury expression levels predict lineage potential and axis-forming ability of in vitro-derived neuromesodermal progenitors
Source: PLoS Biol. 2026 Aug 25;24(8):e3003960. doi: 10.1371/journal.pbio.3003960 (PMC13533433; doi:10.1371/journal.pbio.3003960)
Supplement: Peer review history

# Author comment: Author response to Decision Letter 0

**DOI:** 10.1371/journal.pbio.3003960.r001

5 Dec 2025

Attachment Submitted filename: 162119_1_rebuttal_3597674_t6pnyx.docx

# Editor report: Decision Letter 0

**DOI:** 10.1371/journal.pbio.3003960.r002

**Authors:** Lucas Smith

16 Dec 2025

Dear Dr Binagui-Casas,

Thank you for submitting your manuscript entitled "Brachyury expression levels predict lineage potential and axis-forming ability of in vitro derived neuromesodermal progenitors" for consideration as a Research Article by PLOS Biology. We understand that your manuscript has been reviewed at Review Commons and that you have submitted your paper to us with a revision plan.

I have now had a chance to discuss your manuscript and revision plan with my colleagues on the PLOS Biology editorial team and with an Academic Editor with relevant expertise. I am writing to let you know that we are, in principle, interested in considering a revised version of your study that addresses the previous reviews ~as you propose.

However, please note that, before we can issue our revision decision, we need you to complete your submission by providing the metadata that is required for full assessment. To this end, please login to Editorial Manager where you will find the paper in the 'Submissions Needing Revisions' folder on your homepage. Please click 'Revise Submission' from the Action Links and complete all additional questions in the submission questionnaire.

Once your full submission is complete, your paper will undergo a series of checks. After your manuscript has passed the checks we will send you another email, inviting you to complete the revision.

While we think the revision plan is largely appropriate, the Academic Editor has flagged a few areas where we think you will need to go a bit further - and so our next decision will provide a bit of additional editorial guidance that we hope you will take into account as you revise your study.

To provide the metadata for your submission, please Login to Editorial Manager (https://www.editorialmanager.com/pbiology) within two working days, i.e. by Dec 18 2025 11:59PM.

During the process of completing your manuscript submission, you will be invited to opt-in to posting your pre-review manuscript as a bioRxiv preprint. Visit http://journals.plos.org/plosbiology/s/preprints for full details. If you consent to posting your current manuscript as a preprint, please upload a single Preprint PDF.

Feel free to email us at plosbiology@plos.org if you have any queries relating to your submission.

Kind regards,

Luke

Lucas Smith, Ph.D.

Senior Editor

PLOS Biology

lsmith@plos.org

# Author comment: Author response to Decision Letter 1

**DOI:** 10.1371/journal.pbio.3003960.r003

22 Dec 2025

Attachment Submitted filename: 162119_1_rebuttal_3597674_t6pnyx_auresp_1.docx

# Editor report: Decision Letter 1

**DOI:** 10.1371/journal.pbio.3003960.r004

**Authors:** Lucas Smith

6 Jan 2026

Dear Dr Binagui-Casas,

Thank you again for submitting your manuscript "Brachyury expression levels predict lineage potential and axis-forming ability of in vitro derived neuromesodermal progenitors", which was previously reviewed at Review Commons, for consideration at PLOS Biology, and thanks also for now providing the relevant metadata to complete your submission.

I would like to first apologize for the time it has taken us to get back to you, after you completed your metadata. The delay was caused by our office closure over the Holidays (I had thought your paper would make it back to my desk before we went on break, but I see it was held up a bit and I didnt have a chance to send you this decision before my vacation).

As mentioned in my previous email, your paper and revision plan have been assessed by the PLOS Biology editorial team and by an Academic Editor with relevant expertise and we would like to invite you to complete your revision and resubmit your paper to PLOS Biology.

We think that your revision plan is largely appropriate, however please note that our Academic Editor has flagged a few areas where we think you will need to go a bit further, by providing further validation of the role of Sox2 as well as clarifying how the findings relate to prior work. I have appended the Academic Editor's comments below my signature, and encourage you to thoroughly address his/her feedback, as you address the reviewer concerns.

Given the extent of revision needed, we cannot make a decision about publication until we have seen the revised manuscript and your response to the reviewers' comments. Your revised manuscript is likely to be sent for further evaluation by all or a subset of the reviewers from Review Commons.

In addition to these revisions, you will need to complete some formatting changes, which you will receive in a follow up email. A member of our team will be in touch with a set of requests shortly.

We expect to receive your revised manuscript within 3 months. Please email us (plosbiology@plos.org) if you have any questions or concerns, or would like to request an extension.

At this stage, your manuscript remains formally under active consideration at our journal; please notify us by email if you do not intend to submit a revision so that we may withdraw it.

**IMPORTANT - SUBMITTING YOUR REVISION**

Your revisions should address the specific points made by each reviewer. Please submit the following files along with your revised manuscript:

1. A 'Response to Reviewers' file - this should detail your responses to the editorial requests, present a point-by-point response to all of the reviewers' comments, and indicate the changes made to the manuscript.

*NOTE: In your point-by-point response to the reviewers, please provide the full context of each review. Do not selectively quote paragraphs or sentences to reply to. The entire set of reviewer comments should be present in full and each specific point should be responded to individually, point by point.

You should also cite any additional relevant literature that has been published since the original submission and mention any additional citations in your response.

2. In addition to a clean copy of the manuscript, please also upload a 'track-changes' version of your manuscript that specifies the edits made. This should be uploaded as a "Revised Article with Changes Highlighted" file type.

*Re-submission Checklist*

When you are ready to resubmit your revised manuscript, please refer to this re-submission checklist: https://plos.io/Biology_Checklist

To submit a revised version of your manuscript, please go to https://www.editorialmanager.com/pbiology/ and log in as an Author. Click the link labelled 'Submissions Needing Revision' where you will find your submission record.

Please make sure to read the following important policies and guidelines while preparing your revision:

*Published Peer Review*

Please note while forming your response, if your article is accepted, you may have the opportunity to make the peer review history publicly available. The record will include editor decision letters (with reviews) and your responses to reviewer comments. If eligible, we will contact you to opt in or out. Please see here for more details:

https://blogs.plos.org/plos/2019/05/plos-journals-now-open-for-published-peer-review/

*PLOS Data Policy*

Please note that as a condition of publication PLOS' data policy (http://journals.plos.org/plosbiology/s/data-availability) requires that you make available all data used to draw the conclusions arrived at in your manuscript. If you have not already done so, you must include any data used in your manuscript either in appropriate repositories, within the body of the manuscript, or as supporting information (N.B. this includes any numerical values that were used to generate graphs, histograms etc.). For an example see here: http://www.plosbiology.org/article/info%3Adoi%2F10.1371%2Fjournal.pbio.1001908#s5

*Blot and Gel Data Policy*

We require the original, uncropped and minimally adjusted images supporting all blot and gel results reported in an article's figures or Supporting Information files. We will require these files before a manuscript can be accepted so please prepare them now, if you have not already uploaded them. Please carefully read our guidelines for how to prepare and upload this data: https://journals.plos.org/plosbiology/s/figures#loc-blot-and-gel-reporting-requirements

*Protocols deposition*

To enhance the reproducibility of your results, we recommend that if applicable you deposit your laboratory protocols in protocols.io, where a protocol can be assigned its own identifier (DOI) such that it can be cited independently in the future. Additionally, PLOS ONE offers an option for publishing peer-reviewed Lab Protocol articles, which describe protocols hosted on protocols.io. Read more information on sharing protocols at https://plos.org/protocols?utm_medium=editorial-email&utm_source=authorletters&utm_campaign=protocols

Thank you again for your submission to our journal. We hope that our editorial process has been constructive thus far, and we welcome your feedback at any time. Please don't hesitate to contact us if you have any questions or comments.

Sincerely,

Luke

Lucas Smith, Ph.D.

Senior Editor

PLOS Biology

lsmith@plos.org

------------------------------------

COMMENTS FROM THE ACADEMIC EDITOR:

As pointed out by the reviewers, key points of the manuscript are the new reporter line, lineage tracing of individual cells in vitro, the identification of specific thresholds of Tbxt expression that underlie NMP fate choice and the conclusion that Sox2 levels appear not to impact NMP choice. However, the reviewers raise concerns about the validity of these conclusions, their relationship to prior work, as well as their relevance in vivo. The authors have presented a compelling revision plan that will address most of the issues raised. The proposed in vivo analysis will be a very good addition to the manuscript. However, the revision plan should include more expanded validation of the role of Sox2 as well as clarify how the findings relate to prior work. I provide more specific recommendations below:

1.A key conclusion is that Sox2 levels are not well correlated with fate choice, while Tbxt levels are. A crucial issue that needs to be addressed is how this finding relates to prior studies of the mechanism governing NMP fate choice, in particular Koch et al 2017 and Gouti et al 2017. Both of these studies have published putative gene regulatory networks underlying the fate decision. The current manuscript should spell out more explicitly what alternative mechanism/ interaction network their findings suggest. The authors seem to hint at the possibility that Tbxt does not directly repress Sox2, which suggests that instead Sox2 is only (or much more strongly) repressed by Tbx6, representing a refinement of the existing models by Koch and Gouti. A direct test of this hypothesis will greatly strengthen the manuscript. For instance, if Sox2 is not expected to contribute to the fate bias, then the Tbxt-driven bias should be preserved in conditions of RA/SAG. The authors could test this by expanding on the experiment they propose in the revision plan to also include Tbxt-Low populations in the section ‘Neural and lateral plate mesoderm potency of TBXTGFP and SOX2mCh subpopulations’. The figure presented in the revision plan under “mathematical modeling” should also be included in the revised manuscript.

2.The authors should provide a clearer representation of the two thresholds and a clearer distinction of the biased vs committed state as a function of Tbxt level. Fig 5E shows the data related to the first threshold (for bias); the second, mesoderm commitment threshold is not clearly shown – in fact, my understanding is that committed cells are identified as Sox2 negative, rather than having even higher Tbxt levels. The relevance of Tbxt and Sox2 levels should be clarified for both of these transitions.

3.The manner in which the mathematical model is presented and interpreted is currently misleading and needs to be revised. The ‘model’ presented in the manuscript is not mechanistic and does not have predictive value with respect to the role of Tbxt levels on fate choice. What it does is simply infer the differentiation rates from data, in other words this is an alternative way to represent the observed fate choices in the different conditions. Statements such as the one on line 410, that the model is used to determine dependence on levels, or line 444 ‘to gain mechanistic insight’, or line 470 ‘clonal analysis and mathematical modeling’ – are therefore misleading. I would suggest calling it ‘estimation’ or ‘inference’ of differentiation rates from data.

# Author comment: Author response to Decision Letter 2

**DOI:** 10.1371/journal.pbio.3003960.r005

1 Jul 2026

Attachment Submitted filename: RC-2025-03238_Response_To_Reviewers.docx

# Editor report: Decision Letter 2

**DOI:** 10.1371/journal.pbio.3003960.r006

**Authors:** Lucas Smith

15 Jul 2026

Dear Dr Binagui-Casas,

Thank you for your patience while we considered your revised manuscript "Brachyury expression levels predict lineage potential and axis-forming ability of in vitro derived neuromesodermal progenitors" for publication as a Research Article at PLOS Biology. This revised version of your manuscript has been evaluated by the PLOS Biology editors and the Academic Editor.

The Academic Editor is fully satisfied by the revision and has commented that you have successfully addressed the concerns raised in the last round of review. Based on our Academic Editor's assessment of your revision, we are likely to accept this manuscript for publication. However, before we can do so we need you to address a few data and other policy-related requests in a last short revision. These requests are detailed below.

**IMPORTANT - Please address the following editorial requests:

1) TITLE: We think that your title may need a '-' between 'in vitro' and 'derived'. If you agree we suggest that you adjust your title to:

"Brachyury expression levels predict lineage potential and axis-forming ability of in vitro-derived neuromesodermal progenitors"

2) FINANCIAL DISCLOSURES: Please update the financial disclosures statement, in our online editorial manager system, to indicate whether the sponsors or funders played any role in the study design, data collection and analysis, decision to publish, or preparation of the manuscript.

3) ETHICS STATEMENT: Please update your ethics statement, in your methods section, to include the specific national or international regulations/guidelines to which your animal care and use protocol adhered. Please note that institutional or accreditation organization guidelines (such as AAALAC) do not meet this requirement.

4) DATA AVAILABILITY: You may be aware of the PLOS Data Policy, which requires that all data be made available without restriction: http://journals.plos.org/plosbiology/s/data-availability. For more information, please also see this editorial: http://dx.doi.org/10.1371/journal.pbio.1001797

Note that we do not require all raw data. Rather, we ask that all individual quantitative observations that underlie the data summarized in the figures and results of your paper be made available in one of the following forms:

a. Supplementary files (e.g., excel). Please ensure that all data files are uploaded as 'Supporting Information' and are invariably referred to (in the manuscript, figure legends, and the Description field when uploading your files) using the following format verbatim: S1 Data, S2 Data, etc. Multiple panels of a single or even several figures can be included as multiple sheets in one excel file that is saved using exactly the following convention: S1_Data.xlsx (using an underscore).

b. Deposition in a publicly available repository. Please also provide the accession code or a reviewer link so that we may view your data before publication.

>Regardless of the method selected, please ensure that you provide the individual numerical values that underlie the summary data displayed in the following figure panels as they are essential for readers to assess your analysis and to reproduce it:

Fig 1F; Fig 3B,E; Fig 4B-E; Fig 5C-F; Fig 6B-F

Fig S2A-B; Fig S3C-I; Fig S4A-F; Fig S6A-E; Fig S6B

NOTE: the numerical data provided should include all replicates AND the way in which the plotted mean and errors were derived (it should not present only the mean/average values).

>Please also ensure that figure legends in your manuscript include information on where the underlying data can be found, and ensure your supplemental data file/s has a legend.

>Please ensure that your Data Statement in the submission system accurately describes where your data can be found.

5) DATA AVAILABILITY: In addition to providing the individual numerical data for the figure panels listed above, we also ask that you provide the raw FACS and RNA seq data, as this is standard practice in the field.

> Please provide the FCS files and a picture showing the successive plots and gates that were applied to the FCS files, for all FACS data generated in your study. As these files are usually quite big, you can deposit them in the Flow Repository (http://flowrepository.org/) or another data repository (like zenodo).

>Please deposit the raw transcriptomic data to a publicly available repository, like the Gene Expression Omnibus

^Please make sure these datasets are publicly available, and to update your data availability statement with the relevant DOIs and accession numbers.

6) DATA NOT SHOWN: Please note that per journal policy, we do not allow the mention of "data not shown", "personal communication", "manuscript in preparation" or other references to data that is not publicly available or contained within this manuscript. Please either remove mention of these data or provide figures presenting the results and the data underlying the figures.

As you address these items, please take this last chance to review your reference list to ensure that it is complete and correct. If you have cited papers that have been retracted, please include the rationale for doing so in the manuscript text, or remove these references and replace them with relevant current references. Any changes to the reference list should be mentioned in the cover letter that accompanies your revised manuscript.

In addition to these revisions, you may need to complete some formatting changes, which you will receive in a follow up email. A member of our team will be in touch with a set of requests shortly. If you do not receive a separate email within a few days, please assume that checks have been completed, and no additional changes are required.

We expect to receive your revised manuscript within two weeks.

To submit your revision, please go to https://www.editorialmanager.com/pbiology/ and log in as an Author. Click the link labelled 'Submissions Needing Revision' to find your submission record. Your revised submission must include the following:

- a cover letter that should detail your responses to any editorial requests, if applicable, and whether changes have been made to the reference list

- a Response to Reviewers file that provides a detailed response to the reviewers' comments (if applicable, if not applicable please do not delete your existing 'Response to Reviewers' file.)

- a track-changes file indicating any changes that you have made to the manuscript.

NOTE: If Supporting Information files are included with your article, note that these are not copyedited and will be published as they are submitted. Please ensure that these files are legible and of high quality (at least 300 dpi) in an easily accessible file format. For this reason, please be aware that any references listed in an SI file will not be indexed. For more information, see our Supporting Information guidelines:

https://journals.plos.org/plosbiology/s/supporting-information

*Published Peer Review History*

Please note that you may have the opportunity to make the peer review history publicly available. The record will include editor decision letters (with reviews) and your responses to reviewer comments. If eligible, we will contact you to opt in or out. Please see here for more details:

https://plos.org/published-peer-review-history/

*Press*

Should you, your institution's press office or the journal office choose to press release your paper, please ensure you have opted out of Early Article Posting on the submission form. We ask that you notify us as soon as possible if you or your institution is planning to press release the article.

*Protocols deposition*

To enhance the reproducibility of your results, we recommend that if applicable you deposit your laboratory protocols in protocols.io, where a protocol can be assigned its own identifier (DOI) such that it can be cited independently in the future. Additionally, PLOS ONE offers an option for publishing peer-reviewed Lab Protocol articles, which describe protocols hosted on protocols.io. Read more information on sharing protocols at https://plos.org/protocols?utm_medium=editorial-email&utm_source=authorletters&utm_campaign=protocols

Please do not hesitate to contact me should you have any questions.

Sincerely,

Luke

Lucas Smith, Ph.D.

Senior Editor

lsmith@plos.org

PLOS Biology

# Author comment: Author response to Decision Letter 3

**DOI:** 10.1371/journal.pbio.3003960.r007

31 Jul 2026

Attachment Submitted filename: RC-2025-03238_Response_To_Reviewers_auresp_3.docx

# Editor report: Decision Letter 3

**DOI:** 10.1371/journal.pbio.3003960.r008

**Authors:** Lucas Smith

6 Aug 2026

Dear Anahi,

Thank you for the submission of your revised Research Article "Brachyury expression levels predict lineage potential and axis-forming ability of in vitro-derived neuromesodermal progenitors" for publication in PLOS Biology, and thank you for addressing our last editorial requests in this revision. On behalf of my colleagues and the Academic Editor, Anna Kicheva, I am pleased to say that we can in principle accept your manuscript for publication, provided you address any remaining formatting and reporting issues. These will be detailed in an email you should receive within 2-3 business days from our colleagues in the journal operations team; no action is required from you until then. Please note that we will not be able to formally accept your manuscript and schedule it for publication until you have completed any requested changes.

**IMPORTANT: in addition to addressing any production requests to come, we also ask that you update the paragraph starting on line 282, to make the changes that we have discussed over email (around removing mention of the 5-day data, that is not shown, and replacing this with discussion of your 7-day data in Fig 3G).

Please take a minute to log into Editorial Manager at http://www.editorialmanager.com/pbiology/, click the "Update My Information" link at the top of the page, and update your user information to ensure an efficient production process.

PRESS

We frequently collaborate with press offices. If your institution or institutions have a press office, please notify them about your upcoming paper at this point, to enable them to help maximise its impact. If the press office is planning to promote your findings, we would be grateful if they could coordinate with biologypress@plos.org. If you have previously opted in to the early version process, we ask that you notify us immediately of any press plans so that we may opt out on your behalf.

We also ask that you take this opportunity to read our Embargo Policy regarding the discussion, promotion and media coverage of work that is yet to be published by PLOS. As your manuscript is not yet published, it is bound by the conditions of our Embargo Policy. Please be aware that this policy is in place both to ensure that any press coverage of your article is fully substantiated and to provide a direct link between such coverage and the published work. For full details of our Embargo Policy, please visit http://www.plos.org/about/media-inquiries/embargo-policy/.

Thank you again for choosing PLOS Biology for publication and supporting Open Access publishing. We look forward to publishing your study.

Sincerely,

Luke

Lucas Smith, Ph.D.

Senior Editor

PLOS Biology

lsmith@plos.org
